# Brain‐Computer Interface Training Fosters Perceptual Skills to Detect Errors

**DOI:** 10.1002/advs.76153

**Published:** 2026-07-15

**Authors:** Deland H. Liu, Fumiaki Iwane, Minsu Zhang, Leonardo G. Cohen, José del R. Millán

**Affiliations:** ^1^ Chandra Family Department of Electrical and Computer Engineering, Cockrell School of Engineering The University of Texas at Austin Austin Texas USA; ^2^ National Institute of Neurological Disorders and Stroke National Institute of Health Bethesda Maryland USA; ^3^ Department of Neurology, Dell Medical School The University of Texas at Austin Austin Texas USA; ^4^ Department of Biomedical Engineering, Cockrell School of Engineering The University of Texas at Austin Austin Texas USA; ^5^ Mulva Clinic for the Neurosciences The University of Texas at Austin Austin Texas USA

**Keywords:** brain‐computer interface, electroencephalography, error perception, error‐related potentials, perceptual learning

## Abstract

Accurate perception of subtle visuo‐motor errors is essential for perceptual and sensorimotor learning, and supports timely corrective actions in precision‐based task. However, conventional perceptual training, typically based on response‐accuracy feedback, is limited in improving sensitivity to small, subtle errors. While prior approaches have focused on modulating sensory regions to enhance perceptual learning, we propose an alternative approach that targets a cognitive neural marker: the error positivity (Pe), a component of the error‐related potential (ErrP) originating in the anterior cingulate cortex, a key decision‐making region. We hypothesize that the Pe, which reflects conscious awareness of errors, serves as a modifiable neural correlate of error perception. In a five‐day longitudinal study, we show that providing real‐time feedback on the presence or absence of ErrPs during perceptual training accelerates perceptual learning at $3^{\circ }$ errors and enhances perceptual performance at $6^{\circ }$ errors without accelerating the learning rate, relative to behavioral training alone. These behavioral gains were accompanied by increase in Pe amplitude. Together, these findings offer new neurophysiological insights into the mechanisms of error perception, and establish ErrP‐based brain‐computer interface interventions as a promising approach for fostering perceptual learning in domains where detecting subtle errors is critical.

## Graphical abstract

Accurate perception of visuomotor errors underpins motor precision and learning, yet conventional behavioral training fails to improve sensitivity to subtle errors. Real‐time EEG‐based brain‐computer interface feedback targeting the error positivity component enhances perceptual learning of small errors. This introduces a neural engineering approach for strengthening perceptual skills and addressing clinical conditions involving disrupted error monitoring.

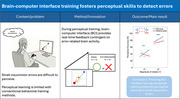

## 1 Introduction

Accurate perception of visuo‐motor errors, particularly when sensory cues are ambiguous or subtle, is critical for effective motor control and learning [1, 2, 3, 4]. Visuo‐motor errors arise when the intended motor commands mismatch the sensory feedback produced by movement [5]. Sensitivity to these discrepancies enables rapid corrective actions, helping prevent accidents or falls [6]. In skilled settings such as surgery, experts must detect and compensate for minute visuo‐motor perturbations to maintain precision, especially in telerobotic environments where delays may exacerbate such errors [7, 8]. Everyday fine motor behaviors, including handwriting and painting, depend similarly on detecting small visuo‐motor deviations [9]. Beyond enabling immediate correction, error detection is a core mechanism of sensorimotor learning: when an error is recognized, the resulting error signals drive adaptive processes that update internal models and refine future motor commands [2, 10, 11].

Perceptual skills are adaptable and can be refined through training, a process known as perceptual learning [12, 13]. However, prior findings indicate that perceptual learning typically follows an asymptotic trajectory, eventually reaching a plateau where further improvements become difficult [12, 14]. This suggests that conventional behavioral perceptual training, which relies on repeated exposure and explicit behavioral feedback, may enhance the detection of large visuo‐motor errors but insufficient for improving sensitivity to smaller errors. Given the importance of small‐errors perception in precision‐dependent tasks, alternative training strategies, potentially leveraging neural engineering approaches, may be required to overcome these limitations.

A promising direction is to target the cognitive and decision‐related neural mechanisms that underlie error perception. In electroencephalogram (EEG) research, the error‐related potential (ErrP) is an event‐related potential (ERP) elicited when individuals perceive an erroneous action either by themselves [15, 16], another person, or an autonomous device [17, 18]. The ErrP is characterised by two distinct components following an incorrect action: the error‐related negativity (ERN), an early negative deflection, and the error positivity (Pe), a subsequent positive deflection, typically observed in the frontal, central and centro‐parietal regions [4, 19, 20, 21, 22]. While the ERN is linked to early error detection [23], the Pe component is associated with conscious error awareness. The Pe amplitude has been shown to be higher during consciously perceived errors compared to those that are partially or not consciously recognized [19, 24]. Additionally, Pe is believed to encode the strength of internal decision evidence indicating an error has occurred [25] and is modulated by error magnitudes and expectation mismatches [26, 27]. These properties make the Pe a strong candidate neural marker of error perception for small visuo‐motor errors.

**FIGURE 1:**
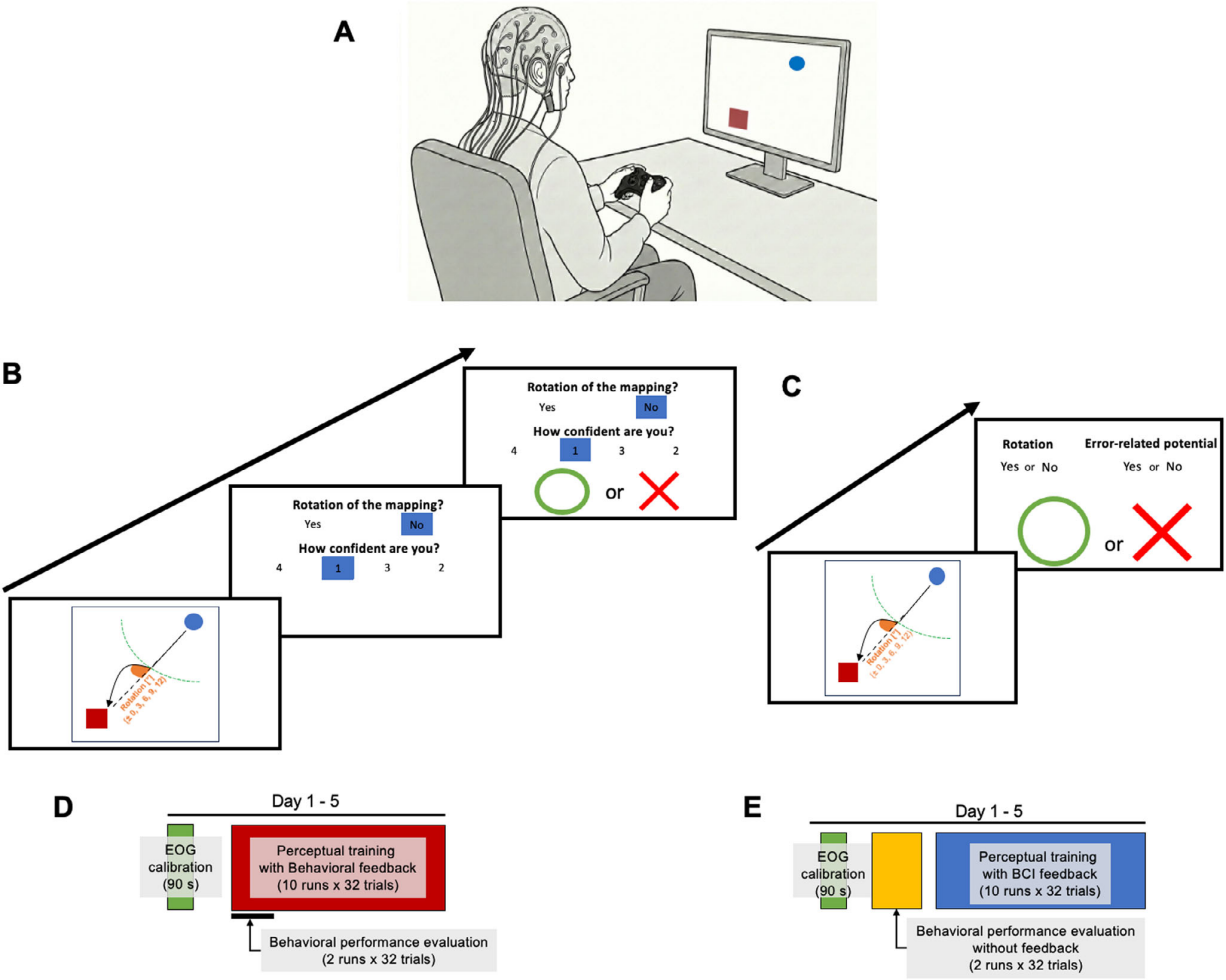
Experimental design. (A) Participants performed a cursor‐reaching task using the left joystick on a gamepad, controlled by their left thumb. (B) In the task, participants were instructed to generate a straight cursor trajectory on each trial, moving the blue circle (cursor) toward the red rectangle (target). The trajectory could be perturbed by a visuomotor rotation. The solid black line illustrates an example cursor trajectory, while the dashed black line represents the ideal straight trajectory. The dashed green curve marks the invisible boundary where the normal joystick‐to‐cursor mapping could be violated by rotations of varying magnitudes ($\pm 0^{\circ }$, $3^{\circ }$, $6^{\circ }$, $9^{\circ }$, $12^{\circ }$). After each trial, participants in the Behavior group indicated whether they perceived a rotation by pressing joystick buttons (blue squares) and received behavioral feedback on their response (a green circle indicating correct response and a red cross indicating incorrect response). (C) Participants in the BCI group completed the same task, but their feedback was the output of their BCI (detection of the presence/absence of an ErrP during the cursor trajectory) together with whether the trial contained a rotation. Feedback was displayed as a green circle when the two were congruent and a red cross when they were incongruent. (D) Participants in the Behavior group underwent 5 days of training, each day consisted of 90 s of EOG calibration followed by 320 trials of perceptual training (10 runs of 32 trials each). **E**: Participants in the BCI group also completed 5 days of training, identical in the number of training trials to the Behavior group, with an additional behavioral assessment of 64 trials (2 runs of 32 trials) conducted after EOG calibration and before the main training block of each day. No feedback was provided during this assessment to prevent learning effects. The BCI group also performed a behavioral assessment block the day after finishing the 5‐day training.

Building on this established evidence, we first hypothesized that the Pe component of the ErrP is a neural correlate of error perception of small visuo‐motor errors and can be modulated through perceptual training. To test this hypothesis, we designed a visuo‐motor error perception task in which participants performed straight cursor‐reaching movements using a joystick (Figure 1A). This task was adapted from established paradigms that use visuo‐motor rotations to investigate ErrPs [1, 4]. In half of the trials, the cursor trajectory was perturbed by visuo‐motor rotations of $3^{\circ }$, $6^{\circ }$, $9^{\circ }$, or $12^{\circ }$. These different magnitudes controlled the difficulty of perception and required corrective actions to maintain a straight path. Participants completed five consecutive days of perceptual training on this task. In Experiment 1, participants received conventional behavioral perceptual training with behavioral feedback on their perceptual decisions (Figure 1B). We hypothesized that such training would be insufficient to improve sensitivity to the smallest visuo‐motor errors ($3^{\circ }$ and $6^{\circ }$). Moreover, we expected that when small errors were missed, Pe amplitudes would be reduced compared to trials in which the same errors were successfully detected. To overcome the limitations of behavioral training and enhance perception of small errors, we developed a novel brain‐computer interface (BCI) training strategy that provided real‐time feedback on the presence or absence of ErrPs during perceptual training. We hypothesized that BCI‐based feedback would amplify the Pe component and lead to greater improvements in perceptual sensitivity to small visuo‐motor errors ($3^{\circ }$ and $6^{\circ }$) compared to behavioral feedback across training days and by the final day of training. To test this hypothesis, Experiment 2 involved a new group of participants who performed the same visuo‐motor error perception task while receiving personalized, real‐time ErrP‐based BCI feedback. This feedback indicated the presence or absence of an ErrP, as well as whether the trial contained a visuomotor rotation (Figure 1C). Our results partially supported the proposed hypotheses. For the smallest visuo‐motor errors ($3^{\circ }$), ErrP‐based feedback accelerated perceptual learning relative to behavioral training alone. For larger, yet still small errors ($6^{\circ }$), ErrP feedback led to an overall and final‐day performance advantage without altering the learning rate. Although ErrP feedback did not increase Pe amplitude relative to behavioral training as originally hypothesized, the perceptual improvements associated with the BCI intervention were accompanied by parallel enhancements in Pe amplitude.

## 2 Results

### 2.1 Pe Component Reflects Error Magnitude and Conscious Error Awareness of Small Visuo‐Motor Errors

**FIGURE 2:**
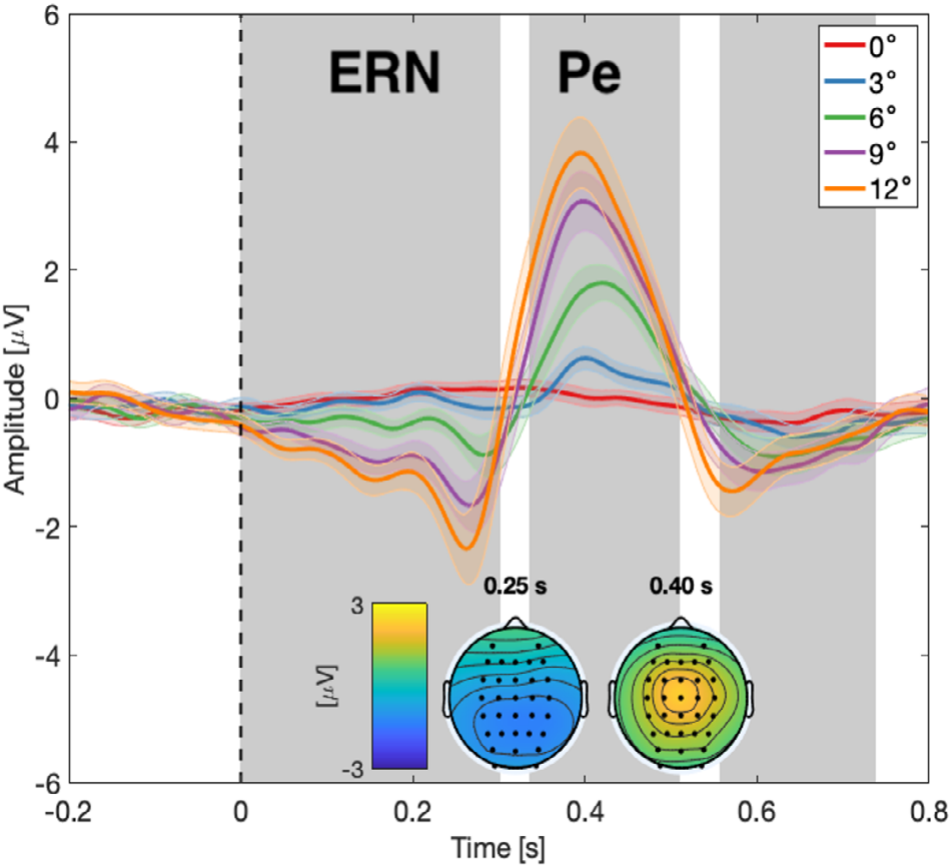
Grand average EEG potentials. Time‐locked grand average EEG potentials at channel Cz of all participants in the two groups (N=32) at different rotation magnitudes ($0^{\circ },3^{\circ },6^{\circ },9^{\circ },12^{\circ }$). Time 0 s, marked by horizontal black dashed line, corresponds to the onset of rotation. The shaded areas around the EEG potentials represent the standard error over participants. Gray shaded regions highlight time intervals where significant differences were found between the error ($3^{\circ },6^{\circ },9^{\circ },12^{\circ }$) and correct class ($0^{\circ }$) epochs. The first region, spanning approx. time window of [0, 300] ms w.r.t trigger onset, is the ERN region. The second region, spanning approx. time window of [340, 520] ms w.r.t trigger onset, is the Pe region. Insets show the topography of EEG amplitude for erroneous epochs (grand average across all subjects and rotation magnitudes) at two time points relative to rotation onset: the ERN (250 ms) and Pe (400 ms).

In Figure 2, the grand‐averaged event‐related potential at Cz computed across all participants from both groups, all training days, and at different rotation magnitudes, shows that the Pe component encodes the magnitudes of errors. Correlation analyses confirmed that Pe amplitude increased progressively with the error magnitude (Behavior group: Spearman correlation, r(69)=0.8509,p<0.0001; BCI group: Spearman correlation, r(70)=0.6897,p<0.0001).

**FIGURE 3:**
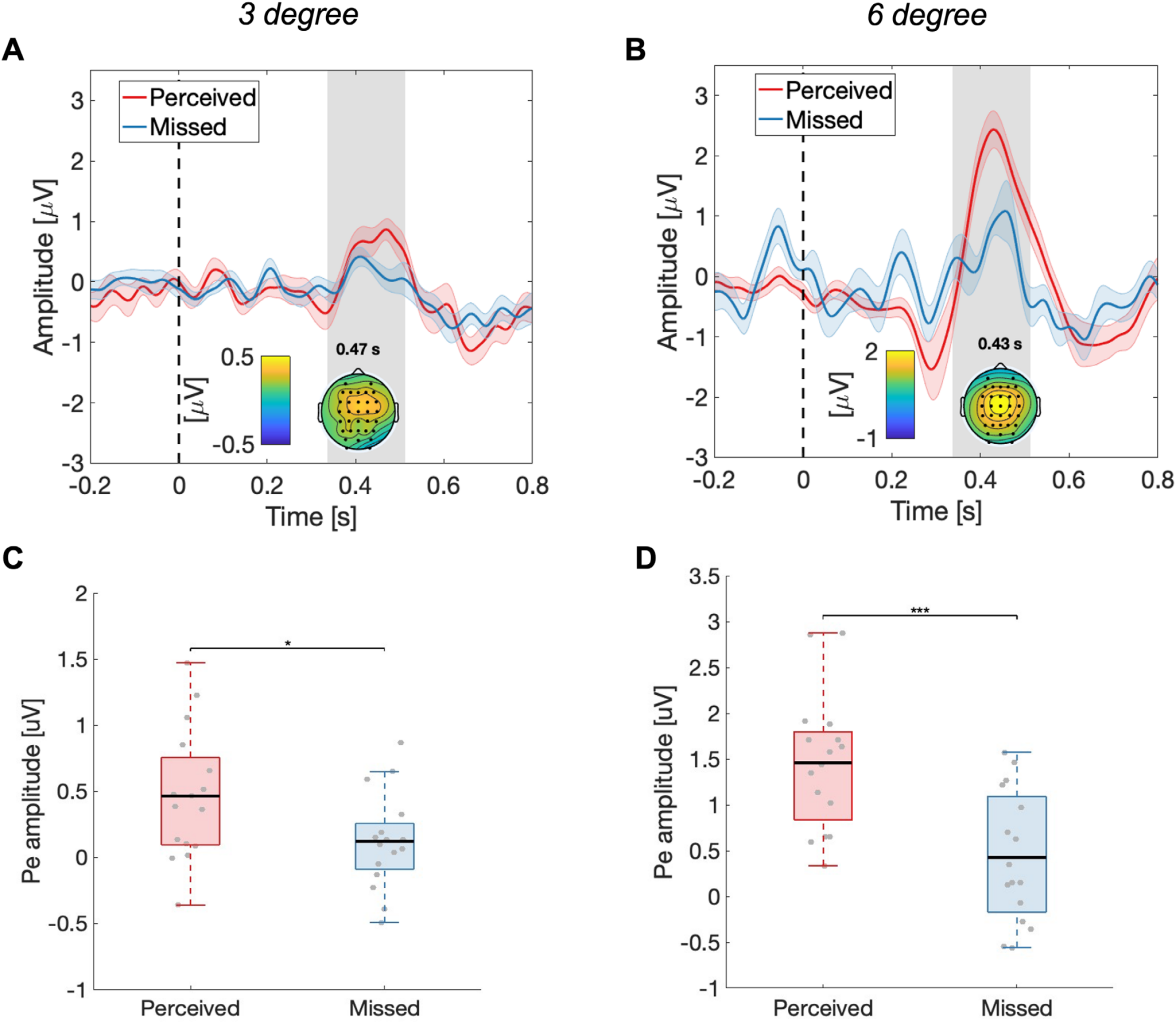
ErrPs for small visuo‐motor errors: comparison of successfully perceived vs missed trials. (A, B) Grand‐averaged ErrPs at Cz for $3^{\circ }$ and $6^{\circ }$ errors in the Behavior group (N=16), shown for successfully perceived trials (red) and the missed conditions (blue). The horizontal dashed black line indicates the onset of rotation. The shaded areas around the EEG potentials represent the standard error across participants. Gray shaded regions mark the Pe region. Insets display EEG amplitude topographies for the two rotations at Pe time points. (C, D) The Pe amplitude at Cz for $3^{\circ }$ and $6^{\circ }$ errors in successfully perceived trials (red) and missed conditions (blue). Each boxplot represents the distribution of the Pe amplitude across participants (N=16), with gray dots representing individual participants' Pe amplitudes. Pe comparisons used paired comparisons. 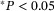, 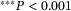.

Figure 3A,B show the grand averaged signals for $3^{\circ }$ and $6^{\circ }$ rotations, comparing successfully perceived trials (“Perceived”) versus trials in which they failed to detect a rotation (“Missed”) within the Behavior group. A clear amplitude difference in the Pe region, indicated by the gray shaded area, was observed between these conditions.

As shown in Figure 3C,D, at $3^{\circ }$ rotations, the Pe amplitudes were significantly higher in successfully perceived trials compared to missed trials and an effect size close to large (Successfully perceived (N = 1195): 0.4651±0.4925 μV, Missed (N = 1995): 0.1212±0.3628 μV, $t\left(15\right)=2.8652,\;p=0.0118,\;Cohen^{\prime }s\;d_{z}=0.7950$). For $6^{\circ }$ rotations, the difference was even more pronounced with a large effect size (Successfully perceived (N = 2521): 1.4605±0.7378 μV, Missed (N = 660): 0.4262±0.7143 μV, $t\left(15\right)=4.5623,\;p=0.0004,\;Cohen^{\prime }s\;d_{z}=1.4245$).

### 2.2 BCI‐Feedback During Perceptual Training Benefits Learning of Small Visuo‐Motor Errors

**FIGURE 4:**
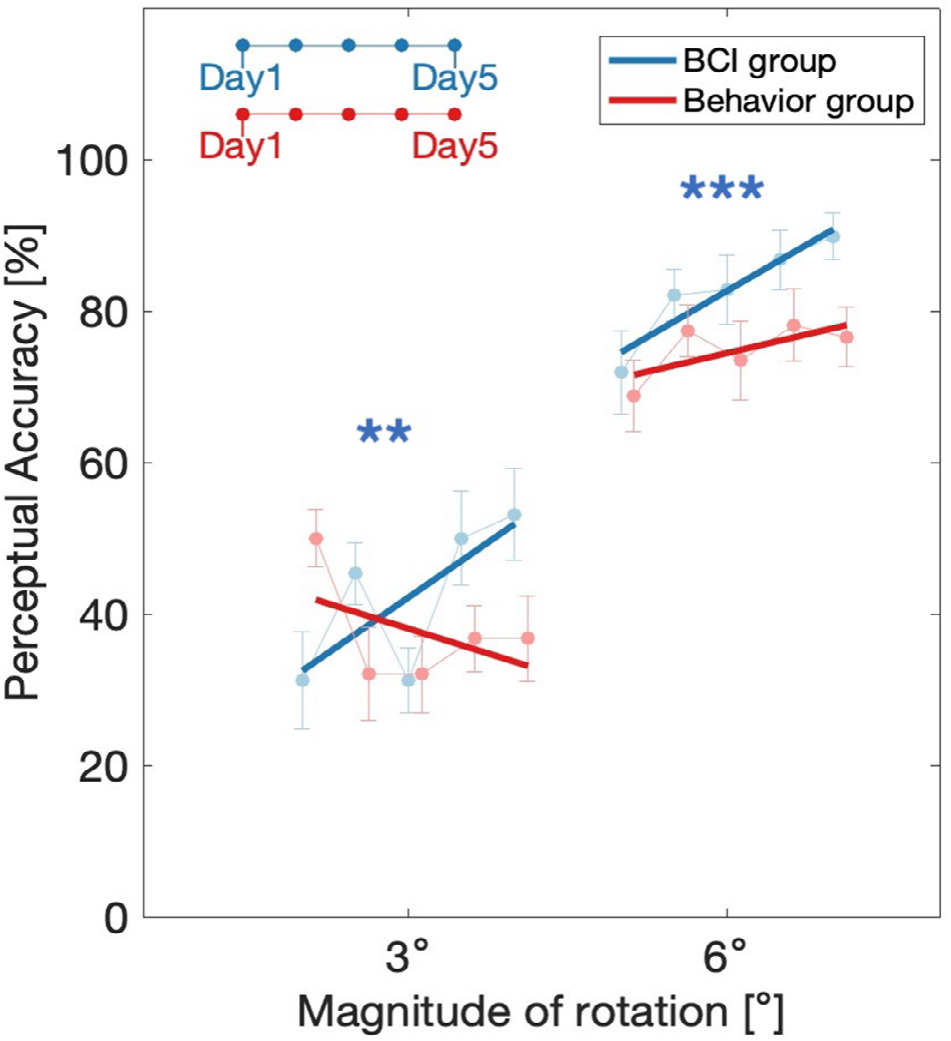
Perceptual accuracies at small rotation magnitudes, across groups, and training days. Perceptual accuracy at the small rotation magnitudes ($3^{\circ }$ and $6^{\circ }$) is shown for the Behavior group (red, N = 16) and the BCI group (blue, N = 16) over the five days of training. Points represent group‐averaged perceptual accuracy, and error bars denote the standard error of the mean. Solid‐lines represent the best‐fit trend generated using the trust‐region algorithm for visualization purposes only and not for statistical testing. Asterisks (*) indicate rotation magnitudes and groups where the fixed effect of training day on perceptual accuracy was significant in the linear mixed‐effects models. 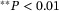 and 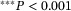.

Figure 4 shows participant's perceptual accuracy at the small rotation magnitudes at $3^{\circ }$ and $6^{\circ }$ across training days in the Behavior (Experiment 1) and BCI groups (Experiment 2). Mixed‐effects modeling of perceptual accuracy at small visuo‐motor error at $3^{\circ }$, with days as the within‐subjects factor and group as the between‐subjects factors, was performed to assess training effects across the 5‐day intervention (see Methods Section 4.11 for details of statistical analyses). Significant days x group from mixed‐effects modeling was observed (F(1,156)=9.7510, p=0.0021). Separate linear mixed‐effects models showed that in the Behavior group, performance declined over days (β(78)=−2.1875±1.5164, F(1,78)=2.0810, p=0.1532, $R^{2}\left(96\right)=0.0847$), while the BCI group exhibited a significant, positive improvement over days (β(80)=4.8437±1.6645, F(1,78)=8.4700, p=0.0047, $R^{2}\left(78\right)=0.1415$), with perceptual accuracy increasing from below chance (50%) to above chance after 3 days and remaining above chance thereafter. At $6^{\circ }$, the Group x Day interaction did not reach significance (F(1,156)=2.1410, p=0.1454). Nonetheless, a significant main effect of group was observed (F(1,156)=4.4336, p=0.0368). In the Behavior group, performance showed an increasing trend over the 5‐days, but did not reach significance (β(78)=1.6406±1.2055, F(1,78)=1.8521, p=0.1775, $R^{2}\left(78\right)=0.1956$). In contrast, the BCI group demonstrated a significant improvement in accuracy and a larger slope coefficient (β(78)=4.0625±1.1342, F(1,78)=12.8300, p=0.0006, $R^{2}\left(78\right)=0.2743$).

**FIGURE 5:**
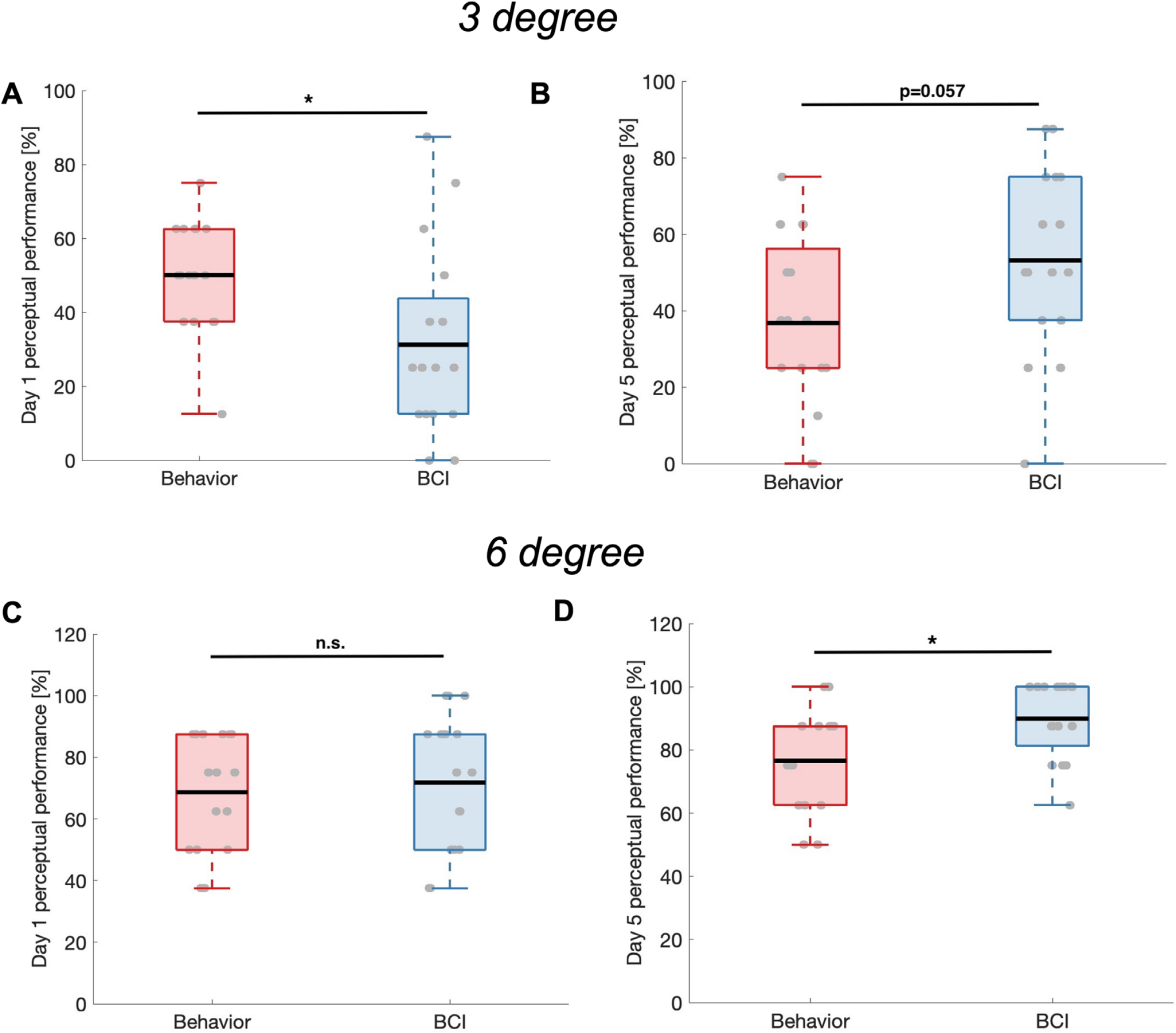
Perceptual accuracy at small rotation magnitudes on Day 1 and Day 5 across groups. (A,B) Comparison of perceptual accuracy at $3^{\circ }$ on the first (Day 1) and final day (Day 5) of training between the Behavior (red, N = 16) and BCI (blue, N = 16) groups, respectively. (B,C): Comparison of perceptual accuracy at $6^{\circ }$ on the first (Day 1) and final day (Day 5) of training between the two groups, respectively. Boxplots show the distribution of perceptual accuracy across participants, with each data point representing an individual participant. Between‐group comparisons used unpaired tests. 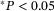, 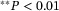.

At $3^{\circ }$, the Behavior group showed a significant, baseline advantage on Day 1 with a large effect size (Behavior: 50.0±15.14%, BCI: 31.25±25.82%; p=0.0146, $d_{z}=0.8900$) (Figure 5A). By Day 5, however, the BCI group had closed this gap and showed higher perceptual accuracy with marginal significance (Behavior: 36.72±22.58%, BCI: 53.13±24.37%; t(30)=1.9800, p=0.0575, $d_{z}=0.7000$) (Figure 5B). For $6^{\circ }$ rotations, the groups performed comparably on Day 1 (Behavior: 68.75±18.82%, BCI: 71.88±22.13%; p=0.6162, $d_{z}=0.1500$) (Figure 5C). By Day 5, the BCI group exhibited significantly higher accuracy with a large effect size (Behavior: 76.56±15.73%, BCI: 89.84±12.26%; p=0.0157, $d_{z}=0.9400$) (Figure 5D).

### 2.3 BCI‐Driven Perceptual Learning Associated with Pe Increases at Small Visuo‐Motor Errors

**FIGURE 6:**
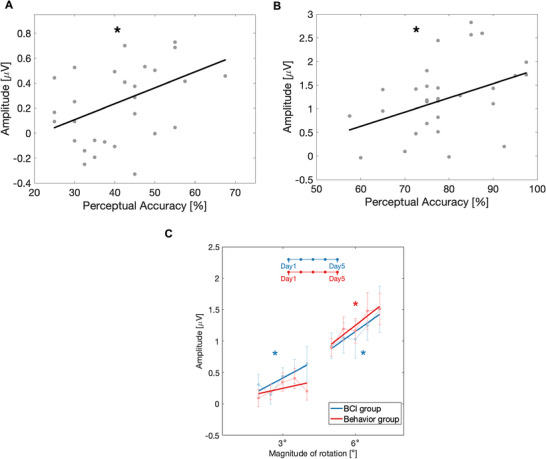
Pe results. (A) Two tailed Spearman correlations between Pe amplitudes at Cz and perceptual accuracy at $3^{\circ }$ across both groups (N = 32). Each data point represents a participant's mean Pe amplitude and perceptual accuracy averaged over the five training days. (B) Same correlation analysis for $6^{\circ }$. (C) Pe amplitude at $3^{\circ }$ and $6^{\circ }$ across the five training days for the Behavior (red, N = 16) and BCI (blue, N = 16) groups. Data points represent the mean Pe amplitude at Cz for each day, with error bars indicating the standard error across participants. Solid lines represent the best‐fit trend generated using the trust‐region algorithm for visualization purposes only and not for statistical testing. Asterisks (*) indicate rotation magnitudes and groups where the fixed effect of training day on Pe was significant in the linear mixed‐effects models. 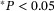.

Correlation analyses at the participant level in Figure 6A,B show that Pe amplitude was significantly and positively correlated with perceptual accuracy at both $3^{\circ }$ (Spearman r(29)=0.3894, p=0.0368) and $6^{\circ }$ (Spearman r(30)=0.3958, p=0.0304) (see Methods Section 4.11 for details of statistical analyses). Group differences in Pe modulation across the training days was then examined using a mixed‐effects model with day as a within‐subject factor and group as a between‐subject factor. This revealed a non‐significant Group × Day interaction at $3^{\circ }$ (F(1,156)=0.9974, p=0.3195). In Figure 6C, within‐group learning trends of Pe at $3^{\circ }$ showed significant increase across days in the BCI group (β(78)=0.1032±0.0468, F(1,78)=4.8657, p=0.0303, $R^{2}\left(80\right)=0.3524$), whereas no significant change was observed in the Behavior group (β(78)=0.0426±0.0387, F(1,78)=1.2075, p=0.2752, $R^{2}\left(78\right)=0.1681$). This pattern mirrors the group‐specific behavioral gains at $3^{\circ }$ (Figure 4). At $6^{\circ }$, the Group × Day interaction was also not significant (F(1,156)=0.0363, p=0.8491), indicating consistent trends in Pe amplitudes between the groups. Subsequent within‐group analysis showed a significant increase in the Pe component over days in both groups (Behavior group: β(78)=0.1508±0.0515, F(1,78)=8.5578, p=0.0045, $R^{2}\left(78\right)=0.4571$; BCI group: β(78)=0.1371±0.0502, F(1,78)=7.4650, p=0.0078, $R^{2}\left(80\right)=0.6833$).

Between‐group comparisons of Pe amplitude revealed no significant differences at Day 5 or Day 1 for either rotation magnitude. At $3^{\circ }$, Day 5 amplitudes were similar (Behavior: 0.1969±0.5509 μV; BCI: 0.6382±0.5578 μV; t(30)=1.43, p=0.1633, $d_{z}=0.5053$), as were baseline values (Behavior: 0.0908±0.5374 μV; BCI: 0.3088±0.6462 μV; t(30)=1.04, p=0.3077, $d_{z}=0.3669$). At $6^{\circ }$, the groups were likewise comparable at Day 5 (Behavior: 1.5127±1.0013 μV; BCI: 1.5084±1.4642 μV; t(30)=0.322, p=0.7487) and Day 1 (Behavior: 0.8994±0.5636 μV; BCI: 0.9220±0.8207 μV; t(30)=0.091, p=0.9281).

### 2.4 Spatial and Temporal Contributions to ErrP Decoding in the BCI Group

**FIGURE 7:**
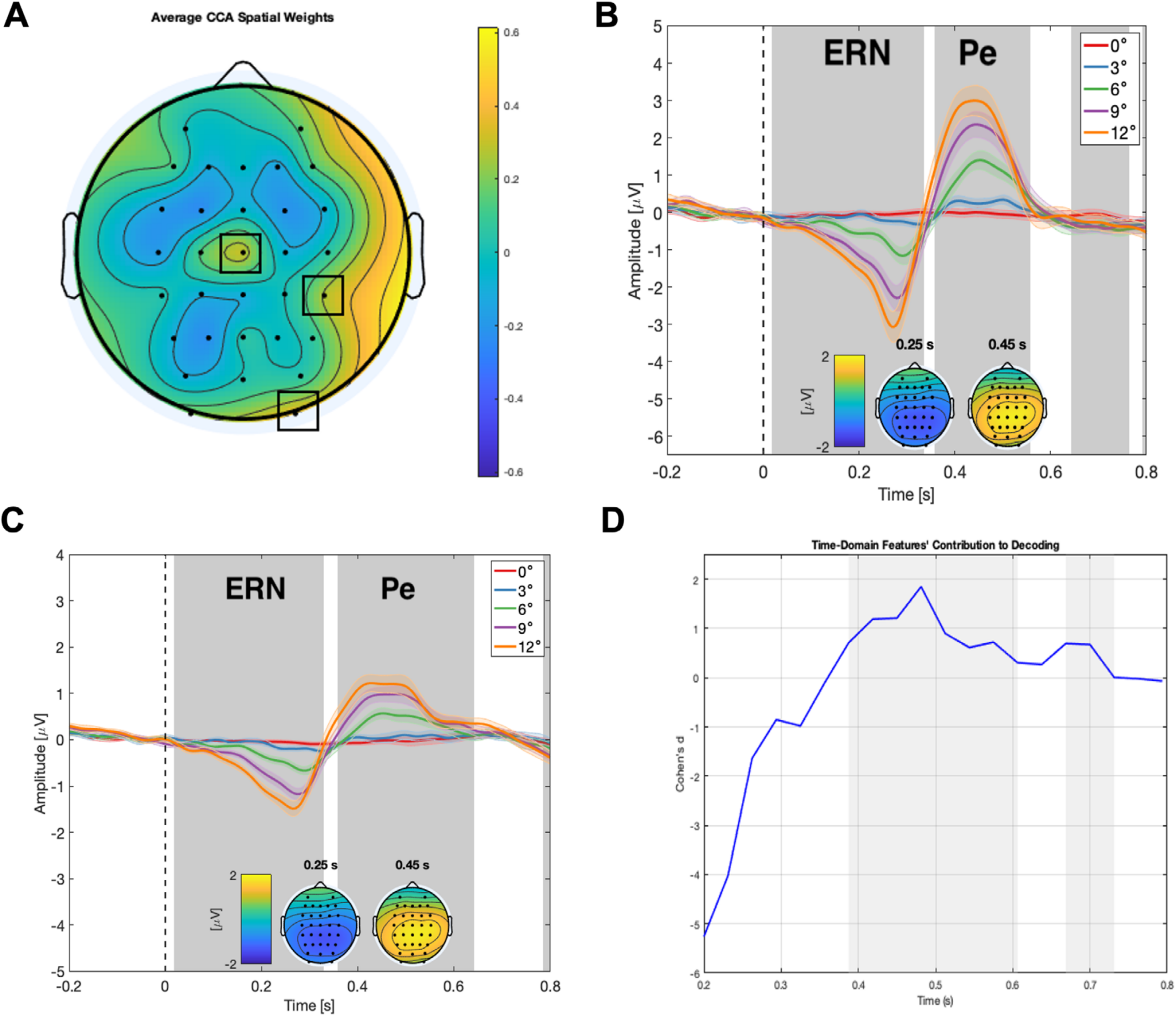
Decoding analyses in the BCI group. (A) Topographical plot of average absolute, z‐scored CCA spatial weights across BCI participants (N = 16). Black squares mark channels with spatial weights significantly greater than zero before correction (Cz, CP4, O2). (B,C): Grand‐average EEG potentials at CP4 and O2 across rotation magnitudes. Time 0 s (black dashed line) indicates rotation onset. Shaded areas show standard error; gray regions highlight intervals where error trials differed significantly from correct trials. Insets show EEG topographies for erroneous epochs at ERN (250 ms) and Pe (450 ms). (D) Effect sizes of absolute, z‐scored time‐domain LDA weights, with the x‐axis denoting timestamps. Gray regions indicate features significantly greater than zero. Spatial and time‐domain weight comparisons were assessed using one‐sample, paired tests against zero. 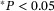, 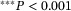.

We examined the spatial and temporal features that contributed most to decoding the presence or absence of ErrPs in the BCI group (see Methods Section 4.10 for details of analyses). Evaluation of z‐scored spatial weights from the canonical correlation analysis identified Cz ($Cohen^{\prime }s\;d_{z}=0.458$), CP4 ($Cohen^{\prime }s\;d_{z}=0.794$), and O2 ($Cohen^{\prime }s\;d_{z}=0.513$) as the channels with the largest effect sizes among the recorded channels and statistically larger than zero (Figure 7A). However, these significances did not survive multiple comparison corrections. Grand‐average waveforms at CP4 and O2 (Figure 7B,C) confirmed physiologically consistent ErrP signatures, with significant differences between error and correct trials in the ERN and Pe time windows. Motor‐area contributions to ErrP decoding were minimal, with small or negative effect sizes in C3 ($d_{z}=-0.596$), C4 ($d_{z}=0.116$), FC3 ($d_{z}=-0.396$), FC4 ($d_{z}=-0.194$), and CP3 ($d_{z}=0.062$), indicating that spatial weights in these channels were near or below the overall mean across channels.

In the time domain, a total of nine features exhibited weights significantly different from zero, as shown in Figure 7D. Of these, seven were located within the Pe region identified in Figure 2. All Pe‐region features showed effect sizes greater than moderate, with the highest effect sizes observed in the [418, 481] ms interval, exceeding $Cohen^{\prime }s\;d_{z}>1$. No frequency‐domain analyses were conducted, as PSD features were extracted solely in the [4–8] Hz theta band, which has been shown to characterize ErrPs [21].

## 3 Discussion

Our results first confirmed prior, established evidence that the Pe amplitude of the ErrP is larger for consciously perceived than for missed small visuo‐motor errors [19, 24]. The positive correlation in Figure 6 between Pe amplitude and perceptual accuracy further establishes Pe as a neural marker of error perception for small visuo‐motor distortions. Consistent with this result, Figure S2 shows that Pe amplitude increased alongside perceptual learning. Behaviorally, we observed that conventional training improved detection of larger visuo‐motor errors ($9^{\circ }$ and $12^{\circ }$) but did not enhance perception of smaller errors ($3^{\circ }$ and $6^{\circ }$) across five training days (Figure S3). In contrast, participants receiving ErrP‐based feedback showed accelerated learning at $3^{\circ }$, as reflected in a significant Group × Day interaction, within‐group improvements in the BCI group that were absent in the Behavior group, and superior final‐day performance (Figures 4 and 5). At $6^{\circ }$, although the Group × Day interaction was not significant, the BCI group consistently outperformed the behavior group, exhibited within‐group improvements absent in the behavior group, and achieved higher accuracy by the final day. As shown in Figure S4, reliable decoding accuracies were achieved in the BCI group. Although decoding was more challenging at smaller visuo‐motor errors, classifier performance nevertheless improved across sessions. Lastly, whilst group‐level Pe amplitude did not differ significantly as hypothesized, the perceptual improvements from the BCI intervention at $3^{\circ }$ and $6^{\circ }$ were accompanied by parallel enhancements in Pe amplitude (Figure 6). Notably, the results suggest a trend whereby the benefit of the BCI intervention increases with perceptual difficulty, such that smaller errors yield a greater relative advantage over conventional training. The effects of BCI feedback were most pronounced at smallest visuomotor error of $3^{\circ }$, and were present but attenuated at $6^{\circ }$, where participants already exhibited relatively high baseline performance in the BCI group (68.750±18.819%). This pattern potentially suggests that ErrP‐based feedback is most effective when error perception is weak or near perceptual threshold, whereas its impact is attenuated when errors are more readily perceivable.

Unlike prior neural engineering interventions that aim to enhance perception by directly modulating early sensory processing, our approach targets the decision‐making circuitry underlying perceptual judgments. Previous work, largely focused on sensory‐cortex modulation, has explored neurofeedback paradigms targeting peak alpha frequency at Pz that improved perception in a 3D multiple‐object tracking task [28]. In addition, fMRI‐based real‐time feedback training increased activation in early visual areas and led to stimulus‐specific visual perceptual learning [29]. Occipital transcranial alternating current stimulation (tACS) at 10 Hz has also been shown to improve orientation discrimination [30]. In the auditory domain, alpha‐tACS over temporal, and central regions enhanced near‐threshold perception [31], and vagus nerve stimulation improved auditory discrimination through cholinergic modulation [32]. In contrast, we intervene at the level of post‐sensory decision processes. Activity changes within the anterior cingulate cortex (ACC), a region central to decision making and cognitive control [33, 34], have been linked to improved perceptual learning outcomes [35]. Similarly, late EEG components reflecting post‐sensory decision processes have been associated with perceptual learning [36]. ErrPs, which originate primarily from the ACC [37, 38, 39, 40, 41, 42], therefore offer a compelling and novel neural target for enhancing perceptual learning through decision‐related rather than sensory‐driven mechanisms.

The mechanism underlying our hypothesis was that ErrP‐based feedback would drive operant conditioning, potentially strengthening Pe responses and thereby improving perceptual performance. Operant conditioning is a well‐established principle in both BCI learning [43, 44, 45, 46] and neurofeedback training [47, 48, 49], where contingent feedback enables participants to learn voluntary modulation of neural activity. Evidence from other domains indicates that operant conditioning can modulate evoked responses, including motor‐evoked potentials [50], pain‐related components [51], and slow cortical potentials [52]. These findings support the plausibility that the Pe could be similarly shaped through operant conditioning. In our study, ErrP‐based feedback combined internal error detection with externally provided reinforcement, a structure likely to strengthen error‐processing pathways and modulate Pe amplitude. Consistent with this mechanism, the BCI group exhibited both accelerated perceptual gains and within‐group increases in Pe amplitude. Although between‐group differences in Pe amplitude were not observed, potentially due to high inter‐individual variability and a limited sample size, future studies could test the causal role of the Pe on perception by experimentally manipulating its amplitude using neuromodulatory techniques.

The operant‐conditioning framework guided the design of the BCI feedback, which consisted of both the decoder output and the nature of the trial. While this explicit and contingent mapping is essential for operant learning, it also means that the BCI group received feedback that differed from the Behavior group in both structure and timing (e.g., no requirement for perceptual response and faster feedback delivery). As such, the current design does not allow us to determine whether the observed improvements are driven solely by feedback contingency on ErrP activity or are partly influenced by differences in feedback structure and exposure. However, co‐modulation of perceptual performance at small error magnitudes of $3^{\circ }$/$6^{\circ }$ and Pe amplitude was observed only in the BCI group (Figures 4 and 6). This pattern may be consistent with ErrP‐contingent operant conditioning and is less readily explained by differences in feedback structure or timing alone. Future studies could incorporate a sham‐BCI condition that preserves the appearance and timing of feedback while removing its dependence on ErrP activity, thereby enabling a more precise isolation of contingency‐driven effects.

It is interesting to note that Figure 2 shows that both ERN and Pe scale with error magnitude. This is consistent with prior findings demonstrating that ERN is linked to high‐level performance monitoring and increases with saliency and the magnitude of the error [26, 53, 54, 55]. These components reflect different stages of error processing: the ERN is associated with rapid detection of mismatch, whereas the Pe is linked to conscious error awareness and perceptual evaluation [25, 56, 57]. The observed scaling of both components suggests that larger visuomotor perturbations amplify both early and later stages of error processing. In addition, in Figure 3, at $3^{\circ }$ rotations, the ERN amplitudes difference was not significant between successfully perceived trials compared to missed trials (Successfully perceived (N = 1195): −0.1241±0.2643 μV, Missed (N = 1995): −0.1195±0.2755 μV, $t\left(15\right)=0.0762,\;p_{corrected}=0.9402,\;Cohen^{\prime }s\;d_{z}=0.0170$). For $6^{\circ }$ rotations, the difference was significant with a larger than medium effect size (Successfully perceived (N = 2521): −0.6163±0.3331 μV, Missed (N = 660): −0.2414±0.7015 μV, $p_{corrected}=0.0262,\;Cohen^{\prime }s\;d_{z}=0.6828$). These results suggest that while the Pe component is more directly associated with conscious error awareness, the ERN may also contribute to conscious awareness, particularly for larger error magnitudes. One possible interpretation is that stronger early error signals increase the likelihood that an error reaches conscious awareness [58]. Given the focus of this study on perceptual learning, we focus on the Pe component as the primary neural marker of error perception in this study.

Post hoc decoding analyses in Figure 7 further confirmed that the BCI feedback was consistent with the operant‐conditioning mechanism, as it primarily reflected Pe‐related activity. To ensure robust ErrP detection, we used a [200, 800] ms decoding window that captured time‐domain features from both the ERN and Pe components, thereby leveraging their combined contributions for decoding. High decoding reliability is essential for effective operant conditioning [59, 60], as inconsistent feedback weakens the contingent reinforcement. Despite the broad temporal window, the decoding results in Figure 7D revealed that the features most responsible for classification accuracy arose primarily from the Pe interval and none from the ERN. Therefore, the feedback delivered to participants was shaped mainly by Pe‐related activity, aligning with the hypothesized operant‐conditioning mechanism.

Interestingly, the decoding analyses (Figure 7A) showed that, in addition to Cz, channels CP4 and O2 contributed to ErrP decoding. Both channels exhibited ErrP‐like waveforms (Figure 7B,C), with timing of the negative and positive components consistent with ERN and Pe observed at fronto‐central sites. These findings suggest that, although the ErrP is typically associated with ACC activity and fronto‐central scalp regions [21], its expression during visuomotor tasks may extend across a broader network including centro‐parietal and occipital regions. Consistent with this broader spatial involvement, it is notable that the observed negative ERN‐like component of the ErrP at around 250 ms post onset exhibits a more centro‐parietal distribution, with stronger amplitudes over centro‐parietal regions compared to fronto‐central regions such as Cz (Figure 2). These observations likely reflects task‐specific modulation of error‐related processing in the context of visuomotor perturbations. Prior work supports this distributed involvement, with posterior parietal ErrPs and theta‐band modulations reported during visuomotor error processing, corrections and control [4, 61, 62], and parietal circuits implicated in perceptual and visuomotor decision‐making [63, 64, 65, 66]. The involvement of CP4 and O2 may also reflect the right hemisphere's specialization in visuospatial processing [67], particularly within the parietal and temporo‐occipital cortices [68]. In addition, prior work has demonstrated that the ERN is task‐dependent and may differ between discrete, task‐level errors and continuously correctable motor errors [54, 58]. In such continuous visuomotor contexts, ERN responses might be less consistently observed, may be attenuated, or exhibit altered spatial distributions. Therefore, the observed signals are consistent with ErrP‐like activity, but in this task likely reflect a distributed error‐processing response that includes both canonical ErrP mechanisms and additional visuomotor/parietal contributions, resulting in a broader and shifted scalp distribution.

A potential confounding factor in our study is that improvements in joystick maneuverability could have contributed to changes in perceptual performance across days or between groups. Cursor‐reaching duration served as an index of joystick control, as participants who maintained straighter trajectories reached the target more quickly. Straighter trajectories could, in principle, enhance the ability to perceive visuo‐motor perturbations, whereas inefficient or curved trajectories require frequent adjustments, increase movement time, and may make perturbations harder to detect. Our analyses found no significant correlation between the cursor‐reaching duration and perceptual performance (r(28)=−0.1218,p=0.5368). We also found no Group × Day interaction in cursor‐reaching duration (F(1,124)=1.2073, p=0.2740) and no main effect of Group (F(1,124)=1.3903, p=0.2406). Within‐group analyses revealed no significant change in cursor‐reaching duration across training days for either the Behavior group (β(78)=−0.0345±0.0280, F(1,78)=1.5257, $p_{corrected}=0.3292$, $R^{2}\left(80\right)=0.5819$) or the BCI group (β(78)=0.0195±0.0199, F(1,78)=0.9640, $p_{corrected}=0.3292$, $R^{2}\left(80\right)=0.7642$). Together, these results indicate that joystick control skills did not influence perceptual performance and are unlikely to account for the within‐ or between‐group perceptual or physiologically differences observed in our study. Another potential confounding factor is the contribution of motor‐related neural activity to the decoding results. Although motor demands were minimized by restricting control to small thumb movements, such activity could still influence ErrP decoding. However, the decoding analyses (Figure 7A) revealed no systematic motor contribution.

A limitation of this study is the absence of an a priori power calculation. The observed effect sizes of the Group × Session interaction effects on perceptual performances were $\eta_{p}^{2}=0.05$ for $3^{\circ }$ and $\eta_{p}^{2}=0.01$ for $6^{\circ }$, corresponding to moderate and small effects, respectively. These results suggest that while effects at $3^{\circ }$ were detectable in the current sample, the study may have been less sensitive to smaller effects at $6^{\circ }$, which could require larger samples in future work. Additionally, a limitation of the study is the imbalance in gender distribution between groups and the relatively low proportion of female participants. To assess the potential impact of gender on perceptual performances and Pe, we included gender as a fixed factor in the mixed‐effects model (Pe Amplitude/perceptual accuracy ∼ Days x Group x Gender + (1|Subject)). No significant interaction involving gender was observed for perceptual performance at $6^{\circ }$ (F(1,152)=0.2300, $p_{\mathrm{corr}\mathrm{ected}}=0.8240$) or $3^{\circ }$ (F(1,152)=0.0500, $p_{\mathrm{corr}\mathrm{ected}}=0.8240$), nor for Pe amplitude at $6^{\circ }$ (F(1,152)=2.1600, $p_{\mathrm{corr}\mathrm{ected}}=0.1710$) or $3^{\circ }$ (F(1,152)=1.8900, $p_{\mathrm{corr}\mathrm{ected}}=0.1710$). This suggests gender might not have influenced perceptual learning or Pe trends between groups. However, we note that the study may be underpowered to detect such effects given the limited and unbalanced gender distribution. A larger and more gender balanced sample size is needed in future work.

Findings from the present study open avenues for future research and for addressing open questions arising from this work. One promising future direction is the application of the ErrP‐BCI intervention to enhance perceptual performance in real‐world settings. For instance, motorsport drivers, who must rapidly detect and respond to subtle dynamic changes on track, may benefit from the perceptual improvements afforded by this approach [69]. Beyond healthy athletes, future work could examine the translational potential of this intervention for reversing sensory‐perceptual impairments associated with aging [70, 71] and with neuropsychiatric conditions such as bipolar disorder [72] and schizophrenia [73]. Compared to conventional behavioral training, an ErrP‐BCI intervention offers a more efficient means of accelerating perceptual learning, while also providing a safer alternative to pharmacological strategies [74]. In addition to evaluating the generalizability of the findings presented here to larger cohorts, a key future direction is to determine whether the five‐day intervention used here produces lasting improvements in perceptual behavior, potentially persisting over weeks or months. Prior work has shown that EEG‐based neurofeedback can produce longer‐term changes in cognitive function and physiology, although these effects have not been specifically examined in the context of BCI‐based perceptual learning. For example, [75] demonstrated that theta/low‐beta neurofeedback training improved episodic and semantic memory one week after training. Similarly, in [76], an alpha neurofeedback study reported that alpha power continued to increase in the training group relative to controls at a follow‐up approximately four months post‐training. In addition, it will be important to investigate whether perceptual performance at small error magnitudes continues to improve with extended training duration, or whether performance reaches a plateau. In the BCI group, at $3^{\circ }$, the early‐phase slope was negligible (β=0.0000±3.4793), whereas the late‐phase slope was positive (β=3.1250±7.9288), indicating that improvement emerged primarily during the later phase of training. At $6^{\circ }$, the BCI group showed positive slopes in both the early phase (β=5.4688±2.7587) and late phase (β=3.5156±2.3044), suggesting continued improvement across the full training period. The presence of positive slopes in the late phase for both error magnitudes might indicate that performance had not plateaued by Day 5. In contrast, the Behavior group showed less consistent patterns. At $3^{\circ }$, performance decreased during the early phase (β=−8.9844±3.1455) and then increased modestly during the late phase (β=2.3438±3.3194). At $6^{\circ }$, the control group showed smaller positive slopes in both the early phase (β=2.3438±2.9112) and late phase (β=1.5625±2.7110). These results suggest weaker and less stable improvement compared to the BCI group. These findings suggest that learning in the BCI group and at $6^{\circ }$ in the Behavior Group is progressive and had potentially not reached a plateau within the current training duration. Extending the number of training sessions would therefore be valuable in the future to determine whether performance continues to improve or eventually stabilizes. Finally, our demonstration that ErrPs can be modulated through perceptual training (Figure S2) and BCI feedback may be valuable in conditions where ErrPs serve as critical biomarkers, such as adaptive control deficits in schizophrenia [77] or heightened performance monitoring in obsessive‐compulsive disorder [78]. In these contexts, ErrP‐BCI approaches that enhance ErrPs may help restore or strengthen cognitive functions disrupted by these disorders.

## 4 Methods

### 4.1 Participants

This study enrolled 32 able‐bodied, healthy volunteers, divided equally into two groups: the Behavior group (N = 16) and the BCI group (N = 16). The Behavior group consisted of two self‐reported females and 14 self‐reported males, with a mean age of 26 years (SD = 5.0) and 14 right‐handed participants. The BCI group included seven self‐reported females and 9 self‐reported males, with a mean age of 25 years (SD = 4.5), and all participants were right‐handed. All participants reported no history of neurological problems, no current use of psychoactive medication, normal color vision, and normal or corrected‐to‐normal visual acuity. The experimental protocol was approved by the local ethics commission (2020‐03‐0073, Austin, Texas, USA). Participants provided written informed consent, adhering to the Declaration of Helsinki, and received compensation for their study participation. The study protocol is published on ClinicalTrials.gov (NCT05311878) and the CONSORT enrollment flow diagram is provided in Figure S1.

A limitation of the study is that sample size was not calculated a priori. The sample size (N = 32; 16 per group) was determined based on prior, similar longitudinal EEG‐BCI studies, where comparable cohort sizes are used [46, 79, 80]. A prospective calculation could define the primary endpoint as the Days x Group interaction effects at $3^{\circ }$ and $6^{\circ }$. Effect sizes (e.g., partial $\eta_{p}^{2}$) could be estimated from pilot data, and sample size determined with α=0.05 and power = 0.8.

### 4.2 EEG and EOG Acquisition

We recorded 32 EEG and 3 electrooculogram (EOG) signals at 512 Hz using an eego system (ANT Neuro, Netherlands). EEG electrodes were located at AF3, AF4, F3, F1, Fz, F2, F4, FC3, FC1, FCz, FC2, FC4, C3, C1, Cz, C2, C4, CP3, CP1, CPz, CP2, CP4, P3, P1, Pz, P2, P4, PO3, POz, PO4, O1, and O2 in 10/20 international coordinates. The ground electrode was placed on the forehead (AFz) and the reference electrode was placed on the inion (Iz). The three EOG electrodes were placed at above the nasion and below the outer canthi of the eyes, and the ground and reference electrodes were placed on the right and left mastoid (M1 and M2), respectively. To reduce signal contamination, participants were asked to avoid excessive eye movements, blinks, and motor movements during trials. Participants underwent 90 s of recording at the start of each recording session in which they were asked to perform three different kinds of eye movements for 30 s each: (1) clockwise and counter‐clockwise rolling of eyeballs, (2) vertical and horizontal eye movements and (3) repeated eye blinks.

### 4.3 Experimental Procedure

The experimental procedure completed by the BCI and Behavior groups is shown in Figure 1C,D. Participants in the Behavior group visited the laboratory for five consecutive days, each lasting approximately 90 min. Each day began with an EOG calibration run, followed by ten runs of perceptual training of 32 trials each. Participants in the BCI group attended the laboratory for six consecutive days. The first five days followed the same structure as the Behavior group with an EOG calibration run and 10 perceptual training runs of 32 trials each. Additionally, each day began with two extra behavioral assessment runs of 32 trials each. These days lasted approximately 110 min. The final, 6th day comprised of only two behavioral assessment runs, and lasted approximately 30 min. In both groups, EEG data was systematically recorded across all days and runs.

### 4.4 Experimental Setup and Task

In this study, participants completed perceptual training in a cursor‐reaching task, where errors were induced through visuo‐motor rotations'a widely used method known to elicit ErrPs [4, 27, 61]. Participants were seated comfortably in front of a 14‐inch display (ThinkPad X1 Carbon, 2560 x 1440 pixels, 60 Hz refresh rate) showing the interface of the cursor‐reaching task (Figure 1A,B). The visual scene included a cursor, represented by a blue circle (Diameter: 20 pixels, 0.5 cm), and a target, represented by a red square (80 x 80 pixels, 1 cm x 1 cm). The cursor consistently started from the top‐right corner of the display, while the target was positioned at the bottom‐left corner, set at a diagonal distance of 9 cm (800 pixels) from the start. Participants used the left joystick of a DualShock4 gamepad (Sony, Japan) as a simple effector to move the cursor from the start to the target. To reduce motor‐related EEG artifacts, they operated the joystick using minimal left‐thumb movements, thereby limiting interference with the neural processes underlying error perception. Because the task required neither fine motor control nor motor learning, any dominant‐hand advantages were likely inconsequential. The cursor maintained a constant speed of 500 pixels per second (approximately 5.6 cm/s) as long as the joystick was engaged. Participants were instructed to move the cursor in a straight and continuous trajectory. All participants completed a practice run before the first session, and all were able to control the joystick skillfully and move the cursor along a straight trajectory before the experiment began.

In each trial, participants could encounter a potential alteration to the normal joystick‐cursor mapping, resulting in a visuo‐motor error. The visuo‐motor perturbation was introduced by adding an angular offset to the intended joystick direction, producing a visual rotation of the cursor rather than a mechanical disturbance of the joystick hardware. This change was triggered when the cursor crossed an invisible boundary set at 50% of the diagonal distance between the start and goal locations (Figure 1A,B). Participants were not informed about the location of this boundary. The visuo‐motor error had one of four possible magnitudes: $3^{\circ }$, $6^{\circ }$, $9^{\circ }$, or $12^{\circ }$. The visuomotor perturbations were presented in randomized upward (clockwise) and downward (anti‐clockwise) directions, reducing the likelihood that direction‐specific visual responses systematically contribute to the decoding performance. Participants were tasked with detecting this rotation and re‐aligning the cursor to its intended straight path.

In both groups, half of the trials in each training day had a visuo‐motor rotation, evenly distributed across the four rotation magnitudes and presented in a randomized sequence. They were referred to as the “error” trials. The remaining trials, with a $0^{\circ }$ rotation, served as “correct” trials. In the BCI group's behavioral assessment runs, the trials were similarly divided, with half designated as “error” trials and the other half as “correct” trials.

### 4.5 Single‐Trial Procedure

Each trial consisted of the (1) cursor‐reaching phase, where participants used the joystick to reach the goal location and the (2) questionnaire or feedback phase. Depending on the experimental group and the nature of the run, the second phase could include a questionnaire, feedback, or both.

#### 4.5.1 Procedure for the Behavior Group

The sequence of a training trial in the Behavior group is displayed in Figure 1A. Each training trial began with an on‐screen text displaying the trial number, after which participants initiated the trial by pressing the X button on the gamepad. Following a randomized delay between 300 and 1000 ms, the cursor and goal locations appeared, marking the start of the cursor‐reaching phase. Once the cursor reached the goal location, a 400 ms delay was followed by two on‐screen questionnaires. The questionnaires consisted of questions; (1). Rotation of the mapping?: whether a visuo‐motor rotation occurred in the current trial with the choice of Yes or No, and (2). How confident are you?: participants' levels of confidence in the former question with responses on a scale from 1 (least confident) to 4 (most confident). Participants selected their answers using the L1, R1, L2, and R2 buttons on the gamepad. Feedback on their perceptual decision was presented 500 ms later, using a green circle (Diameter: 5 cm, 200 pixels) for correct perceptions and a pink X mark (7 cm, 565 pixels per line) for incorrect perceptions. Each run ended with a display of the total number of correctly identified trials. Participants were instructed to achieve the highest possible perceptual accuracy in each run.

#### 4.5.2 Procedure for the BCI Group

The behavioral assessment trials had the same structure as the Behavior group's training trials, with one key difference: no feedback was provided at the end of each trial. This omission ensured that no learning could occur during these runs. Consequently, the BCI group did not receive additional training. Instead, participants were shown only a summary score at the end of each run, indicating the total number of correctly identified trials.

The BCI group's training trials, displayed in Figure 1B, ended with a different kind of feedback after cursor‐reaching. As shown in Figure 1B, the feedback consisted of: (1). Rotation: whether a visuo‐motor error occurred in the current trial with the choice of Yes or No, (2). Error‐related potential: whether the ErrP‐BCI detected the presence of an ErrP in the [200, 800] ms period w.r.t the rotation onset, with the decision Yes or No, and lastly (3) a reward feedback with a green circle (Diameter: 200 pixels, 5 cm) if the decoder output in Error‐related potential aligned with the rotation mapping in Rotation, and a pink X mark otherwise ((7 cm, 565 pixels per line). At the end of each run, participants were shown a performance score representing the number of trials in which the ErrP‐BCI decision correctly matched the presence or absence of rotation.

Before the training runs, it was clearly explained that feedback was provided based on the participant's brain activity. The key instructions were “Please try to achieve the highest possible perception performance in each training run.” and “Whenever you see an incorrect ErrP feedback, please try to assume a mental state/find a strategy where feedback becomes correct in future trials.”

### 4.6 Decoding the Presence/Absence of ErrP in the BCI Group

During perceptual training, feedback for the BCI group included the output of an online, personalized ErrP‐BCI, which detected the presence or absence of an ErrP induced by visuo‐motor rotations. We used a decoder pipeline similar to prior research [27, 81]. We first applied a 4th order casual Butterworth bandpass filter at 1–10 Hz on EEG and removed EOG artifacts using the autocovariance matrix [82]. EEG signals were then segmented into epochs using window of [200, 800] ms relative to the rotation onset of the cursor. As shown in Figure 2, this window captures the most discriminative time‐domain components between the error and correct classes, namely the ERN and Pe components. Epochs with visuo‐motor errors ($3^{\circ }$, $6^{\circ }$, $9^{\circ }$, or $12^{\circ }$) were labeled as 1 (presence of ErrP), and epochs with $0^{\circ }$ as 0 (absence of ErrP). To enhance the signal‐to‐noise ratio (SNR) of the EEG signals, we applied a Canonical Correlation Analysis (CCA)‐based spatial filter to the epochs, retaining only the first three CCA components for dimensionality reduction [83, 84]. Since ErrPs are characterized by specific temporal signatures (i.e., an increase in signal amplitude in the Pe component relative to baseline) and frequency modulations in the theta band (4–10 Hz) [20, 21], we extracted two types of features for each CCA component: (1) downsampled signal amplitudes at 32 Hz (yielding 60 temporal features) and (2) power spectral density (PSD) at 4, 6, 8, and 10 Hz frequency bins calculated using Welch's method (yielding 12 PSD features) [85]. This process produced a feature vector **x** of 72 features per epoch, which was then normalized to the [0, 1] range. The classifier estimated the posterior probability of an ErrP, p(error|x), using a diagonal linear discriminant analysis (LDA), following Equation 1:

(1) $$p\left(error|x\right)=\frac{1}{1+\exp^{-(w^{\prime }x+b)}}$$

ErrPs were identified if p(error|x) exceeded a decision threshold τ [86]. To ensure robustness to non‐stationarities and adapt to changes in ErrP characteristics over time, such as those due to perceptual learning, the BCI classifier and spatial filters, along with τ, were updated after each run using all data from the individual participant. This adaptive updating of the ErrP decoder helped ensure reliability of BCI feedback, which is critical for effective operant conditioning.

To optimize the hyperparameter τ, we performed leave‐one‐run‐out cross‐validation, evaluating τ values from 0 to 1 in increments of 0.025. Since the amplitudes of ErrPs encode the magnitudes of error (Figure 2), classifying between error epochs belonging to small rotations (i.e., $3^{\circ }$) and correct epochs ($0^{\circ }$) can be challenging. To address this, the model trained on each fold was validated on a test fold containing only $0^{\circ }$ correct epochs and $3^{\circ }$ error epochs. The τ value was selected by maximizing the distance from the diagonal (chance line) on the averaged ROC curve and was used in subsequent run [86]. TPs were defined as correctly identified ErrPs in error trials and TNs as correctly identified absence of ErrPs in correct trials. Once the optimal τ was computed, the BCI model was retrained using all available data from the participant and deployed online in the next run.

For the first perceptual training run on Day 1, participants used a generic ErrP decoder built from data collected from all participants in the Behavior group. For this first run, a subject‐specific classifier was not constructed from the assessment runs due to the limited number of trials available (eight trials per rotation magnitude), which might be insufficient for reliable model training. This generic decoder was optimized using leave‐one‐participant‐out cross‐validation to determine its initial τ value.

### 4.7 Computation of Perceptual Accuracy

Perceptual accuracy was defined as the percentage of trials correctly perceived at each rotation magnitude. In the BCI group, these values were obtained from the behavioral assessment runs conducted at the start of each day, which intentionally omitted feedback to prevent learning. Since the Behavior group did not have separate behavioral assessment runs, we matched their initial training trials to the BCI group's assessment trials by day, rotation magnitude, and number of trials and evaluated performance on these initial training trials (Figure 1C,D). For example, if the BCI group completed eight behavioral assessment trials with a $3^{\circ }$ rotation in Day 4, we analyzed the first eight training trials with a $3^{\circ }$ rotation from Day 4 in the Behavior group. This procedure allowed for a direct comparison of perceptual accuracy between groups under equivalent task conditions.

### 4.8 Evaluation of Joystick Handling

To ensure consistent joystick handling across training days and between groups, we computed the average cursor‐trajectory duration for each participant on each day. This duration, measured in seconds, was defined as the time from the appearance of the start and goal locations on the screen until the cursor reached the goal.

### 4.9 Error‐Positivity (Pe) Analysis

To analyze the Pe component of the ErrPs, we first applied a non‐causal, 4th order bandpass Butterworth filter with cutoff frequencies of 1–10 Hz to extract the theta band activity of the EEG signals. EOG artifacts were then regressed out of the EEG signals using the autocovariance matrix of both the EEG and EOG signals, as described by Schlögl et al. [82]. Next, we segmented the pre‐processed EEG signals into epochs using the time window of [0 1000] ms relative to the onset of rotation. An epoch with any sample exceeding 100 μV in any of the recorded channels was considered anomalous and removed. This screening process removed 0.4% of epochs in the Behavior group and 1.4% of epochs in the BCI group. To identify the Pe region, we compared the grand average waveforms of the channel of interest for correct trials ($0^{\circ }$) and error trials ($3^{\circ }$, $6^{\circ }$, $9^{\circ }$, or $12^{\circ }$). The Pe component was defined as the time window where significant paired differences between the grand average waveforms were observed after correction with the Benjamini–Hochberg method. As shown in Figure 2, the Pe component at Cz was observed as a positive deflection within the time window of ∼[340, 520] ms relative to the onset. The amplitude of the Pe component was quantified by measuring the mean voltage within this time frame. EEG data processing and analyses were carried out in Matlab (MathWorks, Natick, MA).

### 4.10 Analyses of Spatial and Temporal Contributions to ErrP Decoding in the BCI Group

To quantify the contribution of individual EEG channels to the ErrP decoder, we extracted the spatial weights from the CCA used as the spatial filtering stage in the decoding pipeline. For each participant, these weights were converted to absolute values, z‐scored, and then averaged across CCA components, runs, and days to obtain a single spatial weight per channel. Averaging across runs was necessary because the decoder and the CCA spatial filters were updated after every run.

To assess the contribution of temporal features, we extracted the LDA weights of the time‐domain features in each run. These weights were also absolute valued and z‐scored across CCA components, runs, and days to produce a single weight for each temporal feature for each participant.

### 4.11 Statistical Analysis

All statistical tests were conducted on data that satisfied their assumptions. Data normality was assessed using the Lilliefors test with a significance threshold of p=0.05 [87].

#### 4.11.1 Primary Behavioral Analyses

The primary behavioral outcome was perceptual accuracy at small visuo‐motor errors of $3^{\circ }\mathrm{and}$ $6^{\circ }$. As planned a priori, we tested whether BCI‐based feedback enhanced perceptual learning relative to behavioral feedback by fitting the following mixed‐effects model:

(2) Perceptual accuracy∼Group×Day+(1|Subject),

where Group (Behavior vs BCI) was a between‐subjects factor, Day (1–5) was a within‐subjects factor, and participants modeled with random intercepts. We reported the Group × Day interaction at 3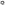 and 6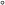. Following the interaction term, two planned contrasts were conducted at each rotation magnitude. First, within‐group learning slopes were evaluated using LME models to test whether perceptual accuracy improved across days within each group:

(3) Perceptual accuracy∼Day+(1|Subject),

Second, between‐group differences at Day 5 and Day 1 were assessed using unpaired two‐tailed t‐tests, as planned a priori. For non‐normal data, Mann–Whitney U tests were applied.

#### 4.11.2 Secondary Behavioral Analyses

Perceptual accuracies at $0^{\circ }$, $9^{\circ }$, and $12^{\circ }$ were analyzed using the same statistical framework but were considered secondary outcomes.

#### 4.11.3 Primary Physiological Analyses

As planned a priori, we examined how perceptual outcome (successfully perceived vs missed) influenced Pe amplitude at $3^{\circ }$ and $6^{\circ }$ in the Behavior group with paired two‐tailed t‐tests. When normality was violated, Wilcoxon signed‐rank tests were used.

Two‐tailed Spearman correlations were used to assess the relationship between Pe amplitude and perceptual performance at $3^{\circ }$ and $6^{\circ }$ at the participant level. For each participant, a single data point was obtained by averaging Pe amplitude and behavioral accuracy across training days at each rotation magnitude. To reduce the influence of extreme observations, we applied robust outlier screening: bivariate outliers were identified using a Mahalanobis distance criterion, and univariate outliers were removed if they exceeded a 3.5 median absolute deviation (MAD) threshold in either variable. The number of observations retained after outlier filtering was recorded and reported for each correlation analysis.

To evaluate trends in Pe amplitude at $3^{\circ }$ and $6^{\circ }$, we performed analyses parallel to those conducted for perceptual accuracy detailed in Section 4.11.1.

#### 4.11.4 Other Secondary Analyses

For the BCI group, spatial contributions to ErrP decoding were assessed by comparing the averaged spatial weights against zero using one‐tailed paired tests. Temporal contributions (LDA feature weights) were evaluated using the same approach.

Two‐tailed Spearman correlations between perceptual performance and cursor‐reaching duration were computed using participant‐level averages across days and rotation magnitudes.

Trends in online decoding accuracy across days at each rotation magnitude were modeled using LME analyses of:

(4) Decoding accuracy∼Day+(1|Subject),

For all paired and unpaired tests, effect sizes were reported using Cohen's $d_{z}$, and t‐statistics were provided when applicable to indicate the magnitude of observed effects. Means and standard deviations were also reported to describe central tendency and variability. All statistical analyses were conducted at the participant level. Statistical significance was set at p<0.05, with 0.05<p<0.10 considered marginally significant and p>0.10 not significant. Effect sizes were interpreted using Cohen's $d_{z}$, with $d_{z}<0.20$ considered small, $0.50\leq d_{z}<0.80$ considered medium, and $d_{z}\geq 0.80$ considered large. LME results were reported using the fixed‐effect coefficient and its standard error, the F‐statistic, corresponding p‐value, and $R^{2}$ as a measure of model fit. All primary analyses were hypothesis‐driven and therefore not corrected for multiple comparisons. Secondary analyses were corrected using the Benjamini–Hochberg procedure.

## Author Contributions

D.L., F.I., and J.d.R.M. conceived and designed the experimental protocols. D.L. and F.I. implemented the experimental protocol. D.L., F.I., and M.Z. were responsible for data acquisition. D.L. and F.I. performed the analyses. D.L., F.I., L.G.C., and J.d.R.M. interpreted results. D.L., F.I., and J.d.R.M. prepared the draft manuscript. All authors reviewed and approved the final version of the manuscript.

## Conflicts of Interest

The authors declare no conflicts of interest.

## Supporting information


**Supporting File**: advs76153‐sup‐0001‐SuppMat.pdf.

## Data Availability

The acquired EEG data and perceptual performance scores for different subjects would be released upon publication (https://zenodo.org/records/20739858).

## References

[1] J. A. Anguera , R. D. Seidler , and W. J. Gehring , “Changes in Performance Monitoring During Sensorimotor Adaptation,” Journal of Neurophysiology 102, no. 3 (2009): 1868–1879. 19605614 10.1152/jn.00063.2009 PMC2746769

[2] S. T. Albert and R. Shadmehr , “The Neural Feedback Response to Error as a Teaching Signal for the Motor Learning System,” Journal of Neuroscience 36, no. 17 (2016): 4832–4845. 27122039 10.1523/JNEUROSCI.0159-16.2016 PMC4846676

[3] L. S. Popa , M. L. Streng , A. L. Hewitt , and T. J. Ebner , “The Errors of Our Ways: Understanding Error Representations in Cerebellar‐Dependent Motor Learning,” The Cerebellum 15, no. 2 (2016): 93–103. 26112422 10.1007/s12311-015-0685-5 PMC4691440

[4] M. Benyamini , I. Demchenko , and M. Zacksenhouse , “Error‐Related EEG Potentials Evoked by Visuo‐Motor Rotations,” Brain Research 1769 (2021): 147606. 34364850 10.1016/j.brainres.2021.147606

[5] R. L. Sainburg and P. K. Mutha , “Error Detection Is Critical for Visual‐Motor Corrections,” Motor Control 20, no. 2 (2016): 187–194. 26314090 10.1123/mc.2015-0022 PMC5364329

[6] D. J. Liss , H. D. Carey , S. Yakovenko , and J. L. Allen , “Young Adults Perceive Small Disturbances to Their Walking Balance Even When Distracted,” Gait & Posture 91 (2022): 198–204. 34740056 10.1016/j.gaitpost.2021.10.019 PMC8671331

[7] C. L. Hewitson , M. J. Crossley , and D. M. Kaplan , “Enhanced Visuomotor Learning and Generalization in Expert Surgeons,” Human Movement Science 71 (2020): 102621. 32452438 10.1016/j.humov.2020.102621

[8] S. Kumar , D. H. Liu , F. S. Racz , et al., “CogniDaVinci: Towards Estimating Mental Workload Modulated by Visual Delays During Telerobotic Surgery—An EEG‐Based Analysis,” in 2023 IEEE International Conference on Robotics and Automation (ICRA) (IEEE, 2023), 6789–6794.

[9] F. Bara and E. Gentaz , “Haptics in Teaching Handwriting: The Role of Perceptual and Visuo‐Motor Skills,” Human Movement Science 30, no. 4 (2011): 745–759. 21272948 10.1016/j.humov.2010.05.015

[10] R. Shadmehr , M. A. Smith , and J. W. Krakauer , “Error Correction, Sensory Prediction, and Adaptation in Motor Control,” Annual Review of Neuroscience 33, no. 1 (2010): 89–108. 10.1146/annurev-neuro-060909-153135 20367317

[11] J. Izawa and R. Shadmehr , “Learning from Sensory and Reward Prediction Errors During Motor Adaptation,” PLOS Computational Biology 7, no. 3 (2011): e1002012. 21423711 10.1371/journal.pcbi.1002012 PMC3053313

[12] A. Karni and D. Sagi , “Where Practice Makes Perfect in Texture Discrimination: Evidence for Primary Visual Cortex Plasticity,” Proceedings of the National Academy of Sciences 88, no. 11 (1991): 4966–4970. 10.1073/pnas.88.11.4966 PMC51788 2052578

[13] J. I. Gold and T. Watanabe , “Perceptual Learning,” Current Biology 20, no. 2 (Jan. 2010): R46–R48. 10.1016/j.cub.2009.10.066 PMC3821996 20129034

[14] E. Zhang and W. Li , “Perceptual Learning Beyond Retinotopic Reference Frame,” Proceedings of the National Academy of Sciences 107, no. 36 (2010): 15969–15974. 10.1073/pnas.1003547107 PMC2936594 20798048

[15] W. J. Gehring , B. Goss , M. G. H. Coles , D. E. Meyer , and E. Donchin , “A Neural System for Error Detection and Compensation,” Psychological Science 4, no. 6 (1993): 385–390.

[16] M. Falkenstein , J. Hoormann , S. Christ , and J. Hohnsbein , “ERP Components on Reaction Errors and Their Functional Significance: A Tutorial,” Biological Psychology 51, no. 2 (2000): 87–107. 10686361 10.1016/s0301-0511(99)00031-9

[17] H. T. van Schie , R. B. Mars , M. G. Coles , and H. Bekkering , “Modulation of Activity in Medial Frontal and Motor Cortices During Error Observation,” Nature Neuroscience 7, no. 5 (2004): 549–554. 15107858 10.1038/nn1239

[18] P. W. Ferrez and J. D. R. Millán , “Error‐Related EEG Potentials in Brain‐Computer Interfaces,” in Toward Brain‐Computing Interfacing , G. Dornhege , J. D. R. Millán , T. Hinterberger , D. J. McFarland , and K.‐R. Müller , Eds. (MIT Press, 2007).

[19] T. Endrass , B. Reuter , and N. Kathmann , “ERP Correlates of Conscious Error Recognition: Aware and Unaware Errors in an Antisaccade Task,” European Journal of Neuroscience 26, no. 6 (2007): 1714–1720. 17880402 10.1111/j.1460-9568.2007.05785.x

[20] R. Chavarriaga and J. D. R. Millán , “Learning from EEG Error‐Related Potentials in Noninvasive Brain‐Computer Interfaces,” IEEE Transactions on Neural Systems and Rehabilitation Engineering 18, no. 4 (Aug. 2010): 381–388. 20570777 10.1109/TNSRE.2010.2053387

[21] R. Chavarriaga , A. Sobolewski , and J. D. R. Millán , “Errare Machinale Est: The Use of Error‐Related Potentials in Brain‐Machine Interfaces,” Frontiers in Neuroscience 8 (2014): 208. 25100937 10.3389/fnins.2014.00208 PMC4106211

[22] D. H. Liu , J.‐C. Hsieh , H. Alawieh , et al., “Novel AIRTrode‐Based Wearable Electrode Supports Long‐Term, Online Brain‐Computer Interface Operations,” Journal of Neural Engineering 22, no. 1 (2025): 016002. 10.1088/1741-2552/ad9edf 39671787

[23] D. M. Olvet and G. Hajcak , “The Error‐Related Negativity (ERN) and Psychopathology: Toward an Endophenotype,” Clinical Psychology Review 28, no. 8 (December 2008): 1343–1354. 18694617 10.1016/j.cpr.2008.07.003 PMC2615243

[24] S. Nieuwenhuis , K. R. Ridderinkhof , J. Blom , G. P. Band , and A. Kok , “Error‐Related Brain Potentials Are Differentially Related to Awareness of Response Errors: Evidence from an Antisaccade Task,” Psychophysiology 38, no. 5 (2001): 752–760. 11577898

[25] M. Steinhauser and N. Yeung , “Decision Processes in Human Performance Monitoring,” Journal of Neuroscience 30, no. 46 (2010): 15643–15653. 21084620 10.1523/JNEUROSCI.1899-10.2010 PMC3073548

[26] R. Vocat , G. Pourtois , and P. Vuilleumier , “Parametric Modulation of Error‐Related ERP Components by the Magnitude of Visuo‐Motor Mismatch,” Neuropsychologia 49, no. 3 (2011): 360–367. 21182849 10.1016/j.neuropsychologia.2010.12.027

[27] F. Iwane , A. Sobolewski , R. Chavarriaga , and J. D. R. Millán , “EEG Error‐Related Potentials Encode Magnitude of Errors and Individual Perceptual Thresholds,” iScience 26, no. 9 (2023): 107524. 37636067 10.1016/j.isci.2023.107524 PMC10448161

[28] B. Parsons and J. Faubert , “Enhancing Learning in a Perceptual‐Cognitive Training Paradigm Using EEG‐Neurofeedback,” Scientific Reports 11, no. 1 (2021): 1–10. 33602994 10.1038/s41598-021-83456-x PMC7892853

[29] K. Shibata , T. Watanabe , Y. Sasaki , and M. Kawato , “Perceptual Learning Incepted by Decoded fMRI Neurofeedback Without Stimulus Presentation,” Science 334, no. 6061 (December 2011): 1413–1415. 22158821 10.1126/science.1212003 PMC3297423

[30] Q. He , X.‐Y. Yang , B. Gong , K. Bi , and F. Fang , “Boosting Visual Perceptual Learning by Transcranial Alternating Current Stimulation over the Visual Cortex at Alpha Frequency,” Brain Stimulation 15, no. 3 (2022): 546–553. 35278689 10.1016/j.brs.2022.02.018

[31] S. Vogeti , M. Faramarzi , and C. S. Herrmann , “Alpha Transcranial Alternating Current Stimulation Modulates Auditory Perception,” Brain Stimulation 16, no. 6 (2023): 1646–1652. 37949295 10.1016/j.brs.2023.11.002

[32] K. A. Martin , E. S. Papadoyannis , J. K. Schiavo , et al., “Vagus Nerve Stimulation Recruits the Central Cholinergic System to Enhance Perceptual Learning,” Nature Neuroscience 27, no. 11 (2024): 2152–2166. 39284963 10.1038/s41593-024-01767-4 PMC11932732

[33] M. E. Walton , P. L. Croxson , T. E. Behrens , S. W. Kennerley , and M. F. Rushworth , “Adaptive Decision Making and Value in the Anterior Cingulate Cortex,” NeuroImage 36 (2007): T142–T154. 17499161 10.1016/j.neuroimage.2007.03.029 PMC2954047

[34] L. A. Newman , D. J. Creer , and J. A. McGaughy , “Cognitive Control and the Anterior Cingulate Cortex: How Conflicting Stimuli Affect Attentional Control in the Rat,” Journal of Physiology‐Paris 109, no. 1–3 (2015): 95–103. 25051488 10.1016/j.jphysparis.2014.06.004 PMC4298471

[35] T. Kahnt , M. Grueschow , O. Speck , and J.‐D. Haynes , “Perceptual Learning and Decision‐Making in Human Medial Frontal Cortex,” Neuron 70, no. 3 (2011): 549–559. 21555079 10.1016/j.neuron.2011.02.054

[36] J. A. Diaz , F. Queirazza , and M. G. Philiastides , “Perceptual Learning Alters Post‐Sensory Processing in Human Decision‐Making,” Nature Human Behaviour 1, no. 2 (Jan. 2017): 0035.

[37] M. Ullsperger and D. Y. Von Cramon , “Subprocesses of Performance Monitoring: A Dissociation of Error Processing and Response Competition Revealed by Event‐Related fMRI and ERPs,” NeuroImage 14, no. 6 (2001): 1387–1401. 11707094 10.1006/nimg.2001.0935

[38] M. Brázdil , R. Roman , M. Falkenstein , P. Daniel , P. Jurák , and I. Rektor , “Error Processing—Evidence from Intracerebral ERP Recordings,” Experimental Brain Research 146 (2002): 460–466. 12355274 10.1007/s00221-002-1201-y

[39] V. Van Veen and C. S. Carter , “The Anterior Cingulate as a Conflict Monitor: fMRI and ERP Studies,” Physiology & Behavior 77, no. 4–5 (2002): 477–482. 12526986 10.1016/s0031-9384(02)00930-7

[40] M. J. Herrmann , J. Römmler , A.‐C. Ehlis , A. Heidrich , and A. J. Fallgatter , “Source Localization (LORETA) of the Error‐Related Negativity (ERN/Ne) and Positivity (Pe),” Cognitive Brain Research 20, no. 2 (2004): 294–299. 15183400 10.1016/j.cogbrainres.2004.02.013

[41] P. W. Ferrez and J. D. R. Millán , “Error‐Related EEG Potentials Generated During Simulated Brain‐Computer Interaction,” IEEE Transactions on Biomedical Engineering 55, no. 3 (March 2008): 923–929. 18334383 10.1109/TBME.2007.908083

[42] G. Dali , M. Brosnan , J. Tiego , et al., “Examining the Neural Correlates of Error Awareness in a Large fMRI Study,” Cerebral Cortex 33, no. 2 (2023): 458–468. 10.1093/cercor/bhac077 PMC9837605 35238340

[43] N. Birbaumer and L. G. Cohen , “Brain‐Computer Interfaces: Communication and Restoration of Movement in Paralysis,” The Journal of Physiology 579, no. 3 (2007): 621–636. 17234696 10.1113/jphysiol.2006.125633 PMC2151357

[44] S. V. Hiremath , W. Chen , W. Wang , et al., “Brain‐Computer Interface Learning for Systems Based on Electrocorticography and Intracortical Microelectrode Arrays,” Frontiers in Integrative Neuroscience 9 (2015): 40. 26113812 10.3389/fnint.2015.00040 PMC4462099

[45] S. Perdikis and J. D. R. Millán , “Brain‐Machine Interfaces: A Tale of Two Learners,” IEEE Systems, Man, and Cybernetics Magazine 6, no. 3 (2020): 12–19.

[46] H. Alawieh , D. Liu , J. Madera , et al., “Electrical Spinal Cord Stimulation Promotes Focal Sensorimotor Activation That Accelerates Brain‐Computer Interface Skill Learning,” Proceedings of the National Academy of Sciences of the United States of America 122, no. 24 (2025): e2418920122. 40493186 10.1073/pnas.2418920122 PMC12184508

[47] S. M. Silverstein , A. A. Menditto , and P. Stuve , “Shaping Attention Span: An Operant Conditioning Procedure to Improve Neurocognition and Functioning in Schizophrenia,” Schizophrenia Bulletin 27, no. 2 (2001): 247–257. 11354592 10.1093/oxfordjournals.schbul.a006871

[48] R. B. Price , I. M. Greven , G. J. Siegle , E. H. Koster , and R. De Raedt , “A Novel Attention Training Paradigm Based on Operant Conditioning of Eye Gaze: Preliminary Findings,” Emotion 16, no. 1 (2016): 110. 26389646 10.1037/emo0000093 PMC4718871

[49] Y. Jiang , W. Jessee , S. Hoyng , et al., “Sharpening Working Memory with Real‐Time Electrophysiological Brain Signals: Which Neurofeedback Paradigms Work?” Frontiers in Aging Neuroscience 14 (2022): 780817. 35418848 10.3389/fnagi.2022.780817 PMC8995767

[50] A. K. Thompson , G. Fiorenza , L. Smyth , B. Favale , J. Brangaccio , and J. Sniffen , “Operant Conditioning of the Motor‐Evoked Potential and Locomotion in People with and Without Chronic Incomplete Spinal Cord Injury,” Journal of Neurophysiology 121, no. 3 (2019): 853–866. 30625010 10.1152/jn.00557.2018 PMC6442914

[51] R. Lousberg , E. Vuurman , T. Lamers , et al., “Pain Report and Pain‐Related Evoked Potentials Operantly Conditioned,” The Clinical Journal of Pain 21, no. 3 (2005): 262–271. 15818078 10.1097/00002508-200505000-00009

[52] B. Rockstroh , N. Birbaumer , T. Elbert , and W. Lutzenberger , “Operant Control of EEG and Event‐Related and Slow Brain Potentials,” Biofeedback and Self‐Regulation 9 (1984): 139–160. 6509107 10.1007/BF00998830

[53] D. M. Olvet and G. Hajcak , “The Error‐Related Negativity (ERN) and Psychopathology: Toward an Endophenotype,” Clinical Psychology Review 28, no. 8 (2008): 1343–1354. 18694617 10.1016/j.cpr.2008.07.003 PMC2615243

[54] O. E. Krigolson , C. B. Holroyd , G. Van Gyn , and M. Heath , “Electroencephalographic Correlates of Target and Outcome Errors,” Experimental Brain Research 190, no. 4 (2008): 401–411. 18629483 10.1007/s00221-008-1482-x

[55] M. Spüler and C. Niethammer , “Error‐Related Potentials During Continuous Feedback: Using EEG to Detect Errors of Different Type and Severity,” Frontiers in Human Neuroscience 9 (2015): 155. 25859204 10.3389/fnhum.2015.00155 PMC4374466

[56] D. M. Olvet and G. Hajcak , “The Stability of Error‐Related Brain Activity with Increasing Trials,” Psychophysiology 46, no. 5 (2009): 957–961. 19558398 10.1111/j.1469-8986.2009.00848.x

[57] L. Wang , Y. Gu , G. Zhao , and A. Chen , “Error‐Related Negativity and Error Awareness in a Go/No‐Go Task,” Scientific Reports 10, no. 1 (2020): 4026. 32132619 10.1038/s41598-020-60693-0 PMC7055303

[58] I. Demchenko , R. Katz , H. Pratt , and M. Zacksenhouse , “Distinct Electroencephalographic Responses to Disturbances and Distractors During Continuous Reaching Movements,” Brain Research 1652 (2016): 178–187. 27693885 10.1016/j.brainres.2016.09.040

[59] M. Grosse‐Wentrup , D. Mattia , and K. Oweiss , “Using Brain‐Computer Interfaces to Induce Neural Plasticity and Restore Function,” Journal of Neural Engineering 8, no. 2 (2011): 025004. 21436534 10.1088/1741-2560/8/2/025004 PMC4515347

[60] Á. Barbero and M. Grosse‐Wentrup , “Biased Feedback in Brain‐Computer Interfaces,” Journal of NeuroEngineering and Rehabilitation 7 (2010): 1–4. 20659350 10.1186/1743-0003-7-34 PMC2927905

[61] P. Arrighi , L. Bonfiglio , F. Minichilli , et al., “EEG Theta Dynamics Within Frontal and Parietal Cortices for Error Processing During Reaching Movements in a Prism Adaptation Study Altering Visuo‐Motor Predictive Planning,” PLOS ONE 11, no. 3 (2016): e0150265. 26963919 10.1371/journal.pone.0150265 PMC4786322

[62] F.‐A. Savoie , F. Thénault , K. Whittingstall , and P.‐M. Bernier , “Visuomotor Prediction Errors Modulate EEG Activity over Parietal Cortex,” Scientific Reports 8, no. 1 (2018): 12513. 30131580 10.1038/s41598-018-30609-0 PMC6104041

[63] C.‐T. Law and J. I. Gold , “Neural Correlates of Perceptual Learning in a Sensory‐Motor, but Not a Sensory, Cortical Area,” Nature Neuroscience 11, no. 4 (April 2008): 505–513. 18327253 10.1038/nn2070 PMC2424192

[64] S. A. Chowdhury and G. C. DeAngelis , “Fine Discrimination Training Alters the Causal Contribution of Macaque Area MT to Depth Perception,” Neuron 60, no. 2 (October 2008): 367–377. 18957227 10.1016/j.neuron.2008.08.023 PMC2642520

[65] P. K. Mutha , R. L. Sainburg , and K. Y. Haaland , “Critical Neural Substrates for Correcting Unexpected Trajectory Errors and Learning from Them,” Brain 134, no. 12 (2011): 3647–3661. 22075071 10.1093/brain/awr275 PMC3235559

[66] S. A. Coombes , D. M. Corcos , L. Sprute , and D. E. Vaillancourt , “Selective Regions of the Visuomotor System Are Related to Gain‐Induced Changes in Force Error,” Journal of Neurophysiology 103, no. 4 (2010): 2114–2123. 20181732 10.1152/jn.00920.2009 PMC2853269

[67] P. M. Corballis , “Visuospatial Processing and the Right‐Hemisphere Interpreter,” Brain and Cognition 53, no. 2 (2003): 171–176. 14607141 10.1016/s0278-2626(03)00103-9

[68] R. D. Brown and J. Buening , “Visuospatial Cognition,” in Neuroscience of Mathematical Cognitive Development: From Infancy Through Emerging Adulthood , R. D. Brown , Ed. (Springer, 2018), 79–96.

[69] O. Lappi , “The Racer's Mind—How Core Perceptual‐Cognitive Expertise Is Reflected in Deliberate Practice Procedures in Professional Motorsport,” Frontiers in Psychology 9 (2018): 1294. 30150949 10.3389/fpsyg.2018.01294 PMC6099114

[70] K. L. Roberts and H. A. Allen , “Perception and Cognition in the Ageing Brain: A Brief Review of the Short‐ and Long‐Term Links Between Perceptual and Cognitive Decline,” Frontiers in Aging Neuroscience 8 (2016): 39. 26973514 10.3389/fnagi.2016.00039 PMC4772631

[71] Z. Kaposzta , A. Czoch , P. Mukli , et al., “Fingerprints of Decreased Cognitive Performance on Fractal Connectivity Dynamics in Healthy Aging,” GeroScience 46, no. 1 (2024): 713–736. 38117421 10.1007/s11357-023-01022-x PMC10828149

[72] M. F. Joseph , T. W. Frazier , E. A. Youngstrom , and J. C. Soares , “A Quantitative and Qualitative Review of Neurocognitive Performance in Pediatric Bipolar Disorder,” Journal of Child and Adolescent Psychopharmacology 18, no. 6 (2008): 595–605. 19108664 10.1089/cap.2008.064 PMC2768898

[73] D. J. Norton , R. K. McBain , D. Öngür , and Y. Chen , “Perceptual Training Strongly Improves Visual Motion Perception in Schizophrenia,” Brain and Cognition 77, no. 2 (2011): 248–256. 21872380 10.1016/j.bandc.2011.08.003 PMC3195882

[74] V. V. Dias , V. Balanzá‐Martinez , M. Soeiro‐de Souza , et al., “Pharmacological Approaches in Bipolar Disorders and the Impact on Cognition: A Critical Overview,” Acta Psychiatrica Scandinavica 126, no. 5 (2012): 315–331. 22881296 10.1111/j.1600-0447.2012.01910.x

[75] Y.‐H. Tseng , K. Tamura , and T. Okamoto , “Neurofeedback Training Improves Episodic and Semantic Long‐Term Memory Performance,” Scientific Reports 11, no. 1 (2021): 17274. 34446791 10.1038/s41598-021-96726-5 PMC8390655

[76] J. H. Gruzelier , “EEG‐Neurofeedback for Optimising Performance. I: A Review of Cognitive and Affective Outcome in Healthy Participants,” Neuroscience & Biobehavioral Reviews 44 (2014): 124–141. 24125857 10.1016/j.neubiorev.2013.09.015

[77] R. M. Reinhart , J. Zhu , S. Park , and G. F. Woodman , “Synchronizing Theta Oscillations with Direct‐Current Stimulation Strengthens Adaptive Control in the Human Brain,” Proceedings of the National Academy of Sciences 112, no. 30 (2015): 9448–9453. 10.1073/pnas.1504196112 PMC4522782 26124116

[78] T. Endrass , J. Klawohn , F. Schuster , and N. Kathmann , “Overactive Performance Monitoring in Obsessive‐Compulsive Disorder: ERP Evidence from Correct and Erroneous Reactions,” Neuropsychologia 46, no. 7 (2008): 1877–1887. 18514679 10.1016/j.neuropsychologia.2007.12.001

[79] L. Pillette , B. N'kaoua , R. Sabau , B. Glize , and F. Lotte , “Multi‐Session Influence of Two Modalities of Feedback and Their Order of Presentation on MI‐BCI User Training,” Multimodal Technologies and Interaction 5, no. 3 (2021): 12.

[80] S. Kumar , H. Alawieh , F. S. Racz , R. Fakhreddine , and J. D. R. Millán , “Transfer Learning Promotes Acquisition of Individual BCI Skills,” PNAS Nexus 3, no. 2 (2024): 076. 10.1093/pnasnexus/pgae076 PMC10903645 38426121

[81] I. Batzianoulis , F. Iwane , and S. Wei , “Customizing Skills for Assistive Robotic Manipulators: An Inverse Reinforcement Learning Approach with Error‐Related Potentials,” Communications Biology 4, no. 1 (December 2021): 1406. 34916587 10.1038/s42003-021-02891-8 PMC8677775

[82] A. Schlögl , C. Keinrath , D. Zimmermann , R. Scherer , R. Leeb , and G. Pfurtscheller , “A Fully Automated Correction Method of EOG Artifacts in EEG Recordings,” Clinical Neurophysiology 118, no. 1 (2007): 98–104. 17088100 10.1016/j.clinph.2006.09.003

[83] F. Iwane , R. Chavarriaga , I. Iturrate , and J. D. R. Millán , “Spatial Filters Yield Stable Features for Error‐Related Potentials Across Conditions,” in IEEE International Conference on Systems, Man, and Cybernetics (2016): 661–666.

[84] F. Iwane , I. Iñaki , R. Chavarriaga , L. Gheorghe , and J. D. R. Millán , “Invariability of EEG Error‐Related Potentials During Continuous Feedback Protocols Elicited by Erroneous Actions at Predicted or Unpredicted States,” Journal of Neural Engineering 18 (2021): 046044. 10.1088/1741-2552/abfa70 33882461

[85] P. Welch , “The Use of Fast Fourier Transform for the Estimation of Power Spectra: A Method Based on Time Averaging over Short, Modified Periodograms,” IEEE Transactions on Audio and Electroacoustics 15, no. 2 (1967): 70–73.

[86] C. Lopes‐Dias , A. I. Sburlea , and G. R. Müller‐Putz , “Online Asynchronous Decoding of Error‐Related Potentials During the Continuous Control of a Robot,” Scientific Reports 9, no. 1 (2019): 1–9. 31772232 10.1038/s41598-019-54109-x PMC6879530

[87] M. Mendes and A. Pala , “Type I Error Rate and Power of Three Normality Tests,” Pakistan Journal of Information and Technology 2, no. 2 (2003): 135–139.

