# Supplementary material for: Brain‐Computer Interface Training Fosters Perceptual Skills to Detect Errors
Source: Adv Sci (Weinh). 2026 Jul 15;13(55):e76153. doi: 10.1002/advs.76153 (PMC13370092; doi:10.1002/advs.76153)
Supplement: Supplementary file 1 [file ADVS-13-e76153-s001.pdf]

5 Supplementary

5.1 CONSORT flow diagram

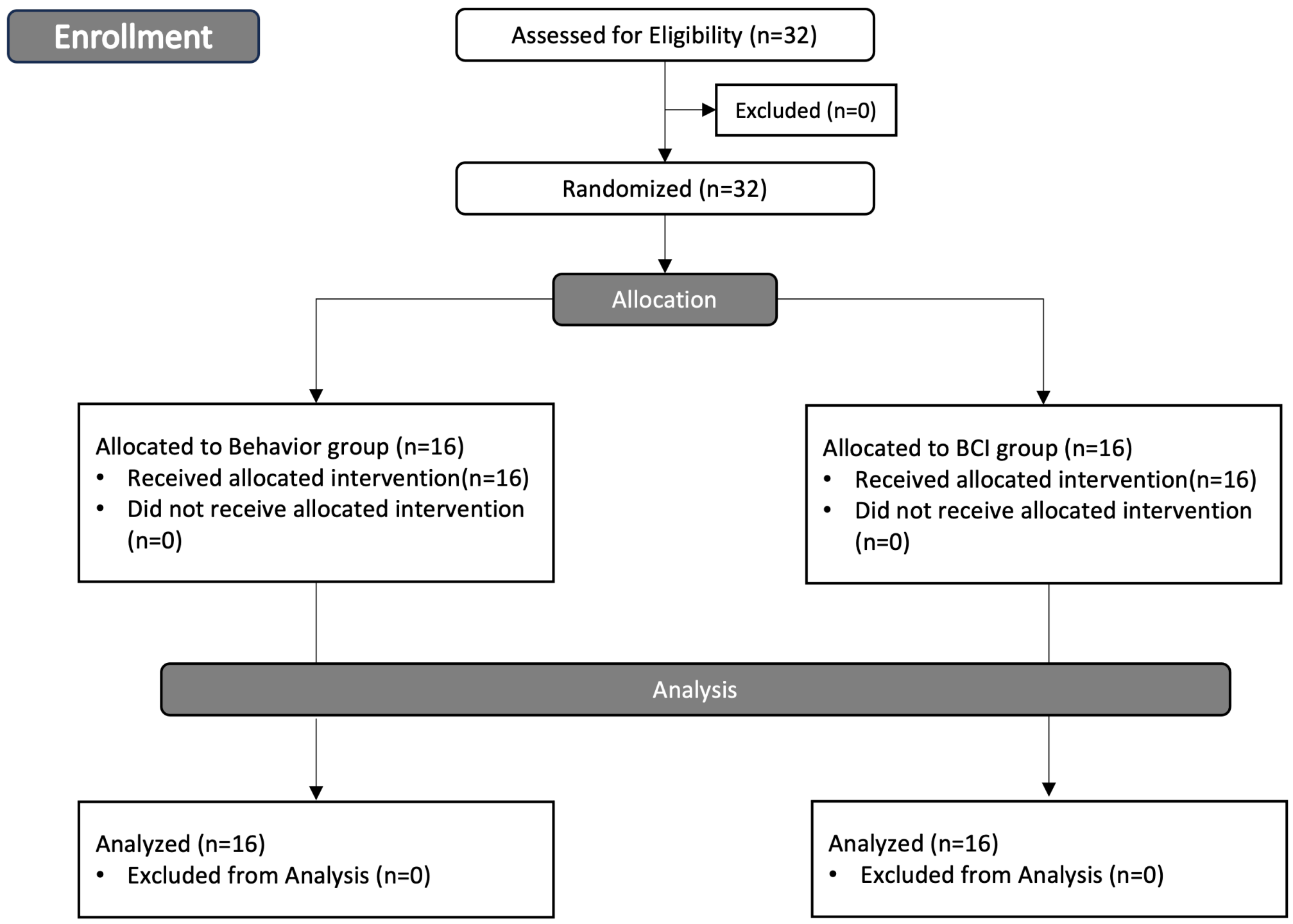

Figure S1: CONSORT flow diagram . Study enrollment diagram. 32 healthy participants were screened and all of them were eligible and agreed to participate. They were randomly assigned to either the Behavior group (N = 16) or the BCI group (N = 16).

5.2 Pe amplified through perceptual training

The trend in Pe amplitude across days in the Behavior group (Experiment 1), as shown in Figure S2, demonstrates that the Pe component can be modulated by perceptual training. Linear mixed-effects analysis of Pe changes across days at each rotation magnitude showed significant enhancements for 6° ($\beta(78) = 0.1508 \pm 0.0515$, $F(1,78) = 8.5578$, $p_{corrected} = 0.0113$, $R^2(80) = 0.4571$), 9° ($\beta(78) = 0.1656 \pm 0.0656$, $F(1,78) = 6.3715$, $p_{corrected} = 0.0227$, $R^2(80) = 0.6601$), and 12° ($\beta(78) = 0.3020 \pm 0.0723$, $F(1,78) = 17.4390$, $p_{corrected} = 0.0004$, $R^2(80) = 0.7752$). Results at 0° approached significance ($\beta(78) = 0.0467 \pm 0.0240$ $F(1,78) = 3.7811$, $p_{corrected} = 0.0692$, $R^2(80) = 0.6495$), although the coefficient was near zero. At 3°, no significant effect was observed ($\beta(78) = 0.0426 \pm 0.0387$, $F(1,78) = 1.2075$, $p_{corrected} = 0.2752$, $R^2(80) = 0.1681$).

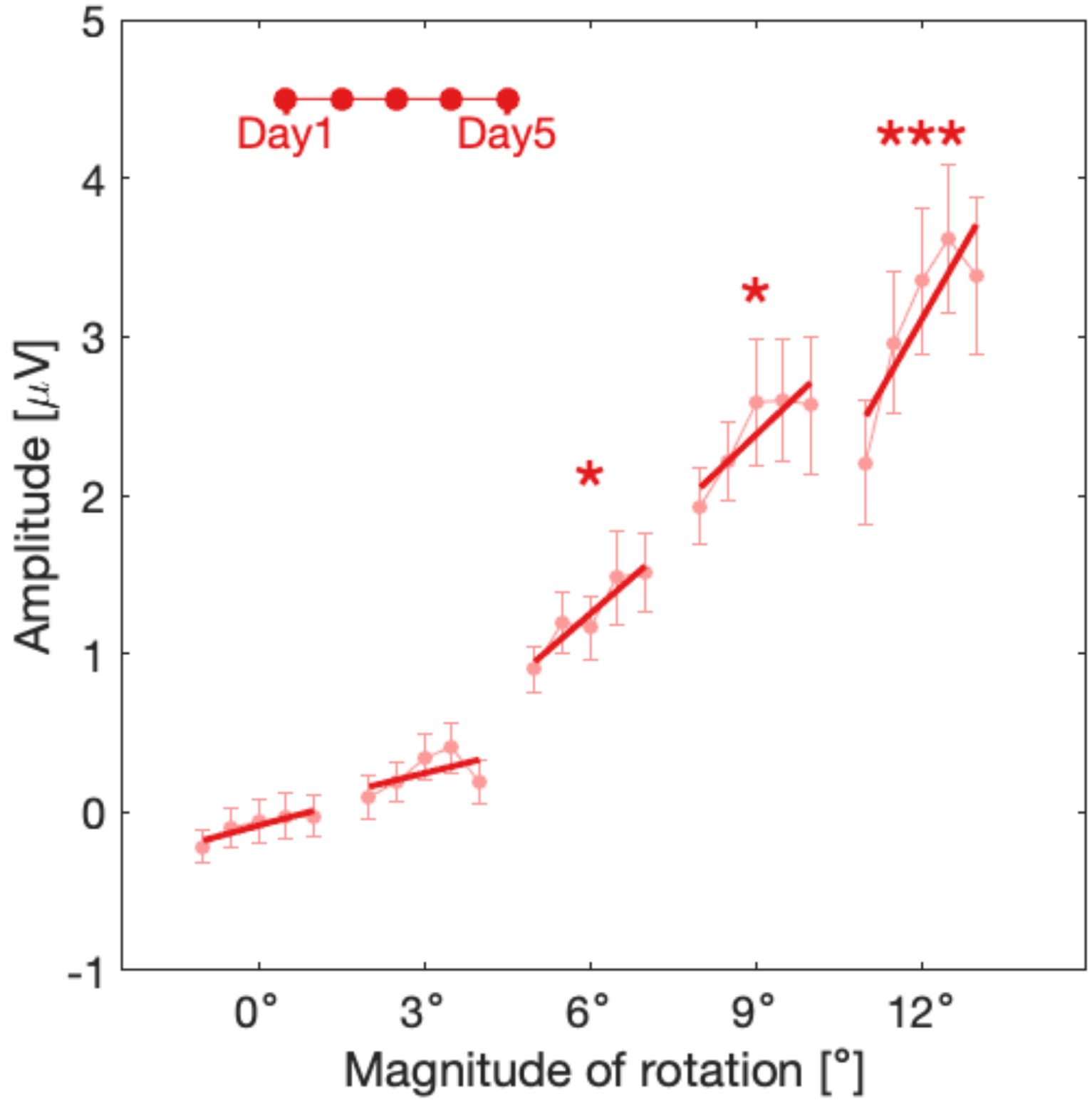

Figure S2: ***Pe amplitudes across days of perceptual training in the Behavior group (Experiment 1).*** Pe amplitude at the Cz electrode for each rotation magnitude and training day in the Behavior group (N=16). Error bars represent the mean and standard error across participants. Lines represent the best-fit trend generated using the trust-region algorithm for visualization purposes only and not for statistical testing. Asterisks (*) indicate rotation magnitudes where the fixed effect of training day on Pe was significant in the linear mixed-effects models. $*P < 0.05$, $***P < 0.001$.

5.3 Individual learning trajectories at 3° and 6°

We quantified individual learning trajectories by fitting a linear regression to each participant's perceptual accuracy across sessions, separately for the 3° and 6° conditions. At 3°, the majority of participants in the BCI group exhibited positive learning slopes (14/16), whereas in the Behavior group a smaller proportion of participants showed positive slopes (7/16). Consistent with this, two-sample t-test showed the mean slope was significantly higher in the BCI group compared to the Behavior group, with a large effect size (Behavior: -0.0218 ± 0.0537, BCI: 0.0484 ± 0.0765; $p = 0.0019$, $d = 1.06$).
At 6°, a similar trend was observed, with more participants showing positive slopes in the BCI group (13/16) compared to the Behavior group (10/16). However, the difference in mean slopes between groups did not reach statistical significance (Behavior: 0.0164 ± 0.0525, BCI: 0.0406 ± 0.0511; $p = 0.1966$, $d = 0.47$).

5.4 Perceptual learning in both groups at 0°, 9° and 12°

Figure S3 shows the perceptual learning trends across all rotation magnitudes tested, including 0°, and larger visuomotor rotations at 9° and 12°. Mixed-effects modeling of perceptual accuracy at 0°, with days as the within-subject factor and group as the between-subjects factor, revealed a marginal days × group interaction ($F(1, 156) = 5.1042$, $p_{corrected} = 0.075$). Both groups improved significantly across days, though the Behavior group showed a steeper slope (Behavior: $\beta(78) = 4.5313 \pm 0.6250$, $F(1, 78) = 52.5620$, $p_{corrected} = 0.0001$,

$R^2(78) = 0.6631$; BCI: $\beta(78) = 2.2266 \pm 0.8062$, $F(1, 78) = 7.6270$, $p_{corrected} = 0.0072$, $R^2(78) = 0.2548$). At 9°, the days × group interaction was not significant ($F(1, 156) = 0.0416$, $p_{corrected} = 1.0000$). Both groups showed significant performance gains with comparable slopes (Behavior: $\beta(78) = 3.2813 \pm 0.8892$, $F(1, 78) = 13.6160$, $p_{corrected} = 0.0001$, $R^2(78) = 0.1454$; BCI: $\beta(78) = 3.0469 \pm 0.7285$, $F(1, 78) = 17.4900$, $p_{corrected} = 0.0002$, $R^2(78) = 0.1794$). Similarly, at 12°, no significant days × group interaction was observed ($F(1, 156) = 0.0000$, $p_{corrected} = 1.0000$). Both groups improved significantly across days with nearly identical slopes (Behavior: $\beta(78) = 1.7969 \pm 0.6038$, $F(1, 78) = 8.8559$, $p_{corrected} = 0.0058$, $R^2(78) = 0.3874$; BCI: $\beta(78) = 1.7969 \pm 0.6200$, $F(1, 78) = 8.3985$, $p_{corrected} = 0.0058$, $R^2(78) = 0.0950$).

Between-group comparisons of Day 5 perceptual accuracy showed no differences at any rotation magnitude. At 0°, no difference between groups were observed (Behavior group: $90.2340 \pm 7.2056\%$, BCI group: $86.5230 \pm 8.0460\%$, $t(30) = 1.3743$, $p_{corrected} = 0.5385$, $Cohen's\ d_z = 0.4859$). No differences were observed at 9° (Behavior: $98.4380 \pm 4.2696\%$, BCI: $98.4380 \pm 4.2696\%$, $p_{corrected} = 1.0000$, $Cohen's\ d_z = 0.0000$) or at 12° (Behavior: $99.2190 \pm 3.1250\%$, BCI: $99.2190 \pm 3.1250\%$, $p_{corrected} = 1.0000$, $Cohen's\ d_z = 0.0000$)

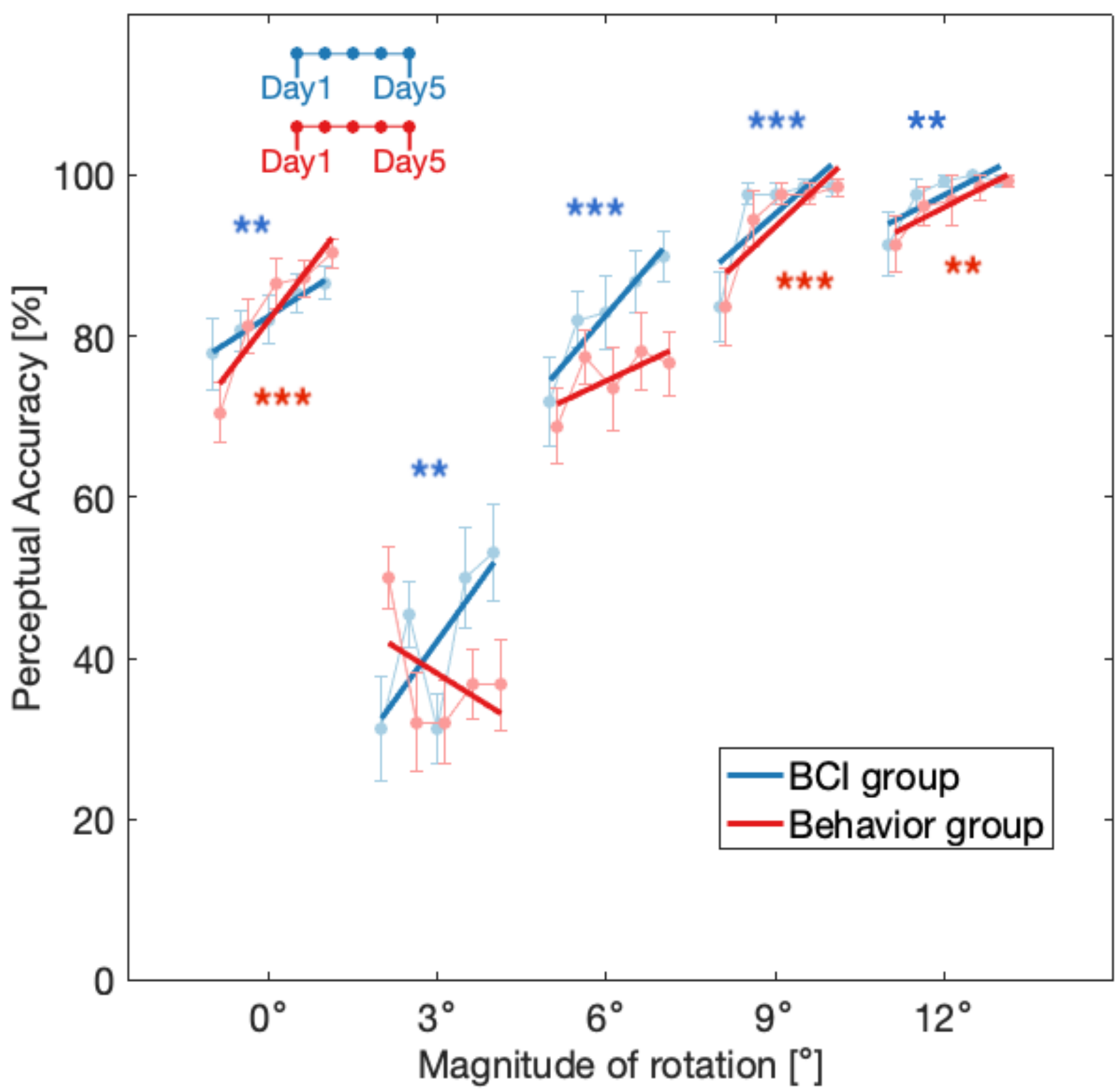

Figure S3: ***Comparison of accuracy between groups across rotation magnitudes and training days.*** Perceptual accuracy at each rotation magnitude ($0°, 3°, 6°, 9°, 12°$) for the Behavior group (red, N=16) and the BCI group (blue, N=16) across the 5 intervention days. The data points show the mean perceptual accuracies across participants, with error bars indicating the standard error. Solid-lines represent the best-fit trend generated using the trust-region algorithm for visualization purposes only and not for statistical testing. Asterisks (*) indicate rotation magnitudes where the fixed effect of training day on perceptual accuracy was significant in the linear mixed-effects models. $**P < 0.01$ and $***P < 0.001$.

5.5 Perceptual learning across six days of training in the BCI group

To evaluate the full effect of training in the BCI group, we examined perceptual accuracy across all six days. The 6-day trends were consistent with the 5-day analysis, showing significant performance increases at all

rotation magnitudes: 0°: $\beta(96) = 2.0759 \pm 0.5749$, $F(1,94) = 13.04$, $p_{corrected} = 0.0023$, $R^2(96) = 0.3212$; 3°: $\beta(96) = 3.8839 \pm 1.2006$, $F(1,94) = 10.47$, $p_{corrected} = 0.0034$, $R^2(96) = 0.1774$; 6°: $\beta(96) = 3.5714 \pm 0.7944$, $F(1,94) = 20.21$, $p_{corrected} = 0.0005$, $R^2(96) = 0.3420$; 9°: $\beta(96) = 2.0982 \pm 0.5286$, $F(1,94) = 15.76$, $p_{corrected} = 0.0015$, $R^2(96) = 0.1410$; 12°: $\beta(96) = 1.3839 \pm 0.4306$, $F(1,94) = 10.33$, $p_{corrected} = 0.0023$, $R^2(96) = 0.0971$).

5.6 Online ErrP decoding performance across training days

Figure S4 shows the BCI's online decoding accuracy of ErrP presence/absence across rotation magnitudes. Decoding accuracy for 0°, 6°, 9°, and 12° consistently remained above chance level (50%) throughout training, while performance at 3° reached chance by Day 2 and remained above it thereafter.

Significant improvements in decoding performance were observed over days for 3°, 6°, 9°, and 12° rotations: 3°: $\beta(78) = 1.7379 \pm 0.8258$, $F(1,78) = 4.4289$, $p_{corrected} = 0.0483$, $R^2 = 0.0525$; 6°: $\beta(78) = 4.2550 \pm 0.6946$, $F(1,78) = 37.5304$, $p_{corrected} = 0.0002$, $R^2 = 0.5630$; 9°: ($\beta(78) = 0.0270 \pm 0.0058$, $F(1,78) = 21.4471$, $p_{corrected} = 0.0002$, $R^2 = 0.5296$; and 12°: $\beta(78) = 3.0781 \pm 0.4764$, $F(1,78) = 41.7476$, $p_{corrected} = 0.0002$, $R^2 = 0.5512$. In contrast, 0° showed a non-significant trend in decoding performance ($\beta(78) = -0.4320 \pm 0.5442$, $F(1,78) = 0.6329$, $p_{corrected} = 0.2000$, $R^2 = 0.0078$). The average FPR remained stable across sessions around 45% throughout the days ($\beta(78) = -0.0043 \pm 0.0054$, $F(1,78) = 0.6302$, $p_{corrected} = 0.4297$, $R^2 = 0.0078$).

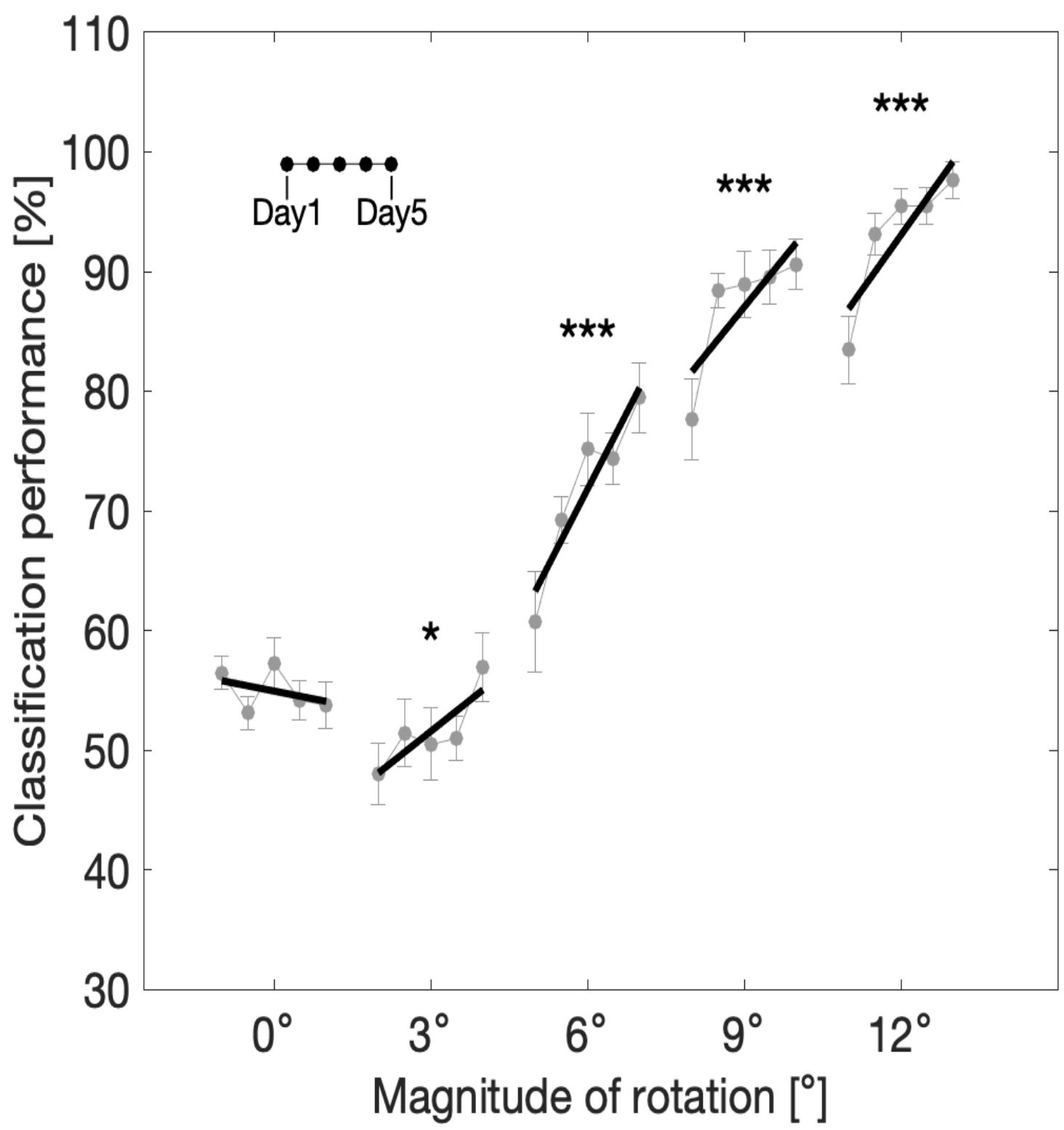

Figure S4: ***Online decoding accuracy of the presence or absence of ErrPs (Experiment 2).*** Online decoding accuracy of the presence or absence of ErrPs at each rotation magnitude ($0°, 3°, 6°, 9°, 12°$) in the BCI group (N=16) across the training days. Data points represent the mean classification performance across participants, with error bars indicating the standard error. Solid-lines represent the best-fit trend generated using the trust-region algorithm for visualization purposes only and not for statistical testing. Asterisks (*) indicate rotation magnitudes where the fixed effect of training day on classification accuracy was significant in the linear mixed-effects models. $*P < 0.05$ and $***P < 0.001$.
